# Histopathological Assessment of Myocardial Ischemia-Reperfusion Injury Using Transformer-Based Artificial Intelligence: Model Comparison Study

**DOI:** 10.2196/80403

**Published:** 2026-06-04

**Authors:** Chengnan Liu, Min Xu, Yanxia Lv, Zhenzhong Zhu, Yifan Pan, Yunxiang Wang

**Affiliations:** 1 Department of Vasculocardiology, Yongkang First People's Hospital Affiliated to Hangzhou Medical College, 599 Jinshan West Road, Dongcheng Street, Yongkang, Zhejiang Province, 321300, China, 86 0579-89279021; 2 Department of Pharmacy, Yongkang First People's Hospital Affiliated to Hangzhou Medical College, Yongkang, Zhejiang Province, China

**Keywords:** artificial intelligence, transformer model, myocardial ischemia-reperfusion, histopathological diagnosis, medical informatics, deep learning

## Abstract

**Background:**

Myocardial ischemia-reperfusion injury (MIRI) poses diagnostic challenges due to complex histopathological changes.

**Objective:**

This study aimed to develop an intelligent framework for evaluating MIRI on hematoxylin-eosin–stained slides, to compare major deep learning architectures, and to determine the advantages of transformer models across multiple interventions and time points.

**Methods:**

A total of 1280 whole-slide images (~62,000 tiles) from public datasets were analyzed across antioxidant, β-blocker, calcium channel blocker, and control groups at 6, 24, and 72 hours. Seven model families (convolutional neural networks, recurrent neural networks, long short-term memory networks, autoencoders, graph convolutional networks, variational autoencoders, and transformers) were trained under unified preprocessing, with generative adversarial networks used exclusively for leakage-free augmentation. Weak supervision used a clustering-constrained attention multiple-instance learning strategy, and segmentation applied a Transformer-UNet. Data were split into 8:1:1 at the subject level, with 5-fold cross-validation.

**Results:**

The transformer achieved the best performance (accuracy=0.942; area under the curve=0.982; and *F*_1_-score=0.958). Segmentation Dice scores were 0.847 (necrosis) and 0.821 (apoptosis). Predictions strongly agreed with expert measurements (*r*=0.886; Bland-Altman limits +5% or −5%), and attention maps aligned with necrotic borders and inflammatory foci. Temporal trends matched biological expectations, with the antioxidant group showing the most stable improvement.

**Conclusions:**

Transformer-based pathology offers accurate, robust, and interpretable assessment of MIRI and provides a scalable framework for dynamic injury quantification and therapeutic evaluation.

## Introduction

Myocardial infarction (MI) is one of the leading causes of disability and mortality worldwide [1]. The widespread adoption of reperfusion therapies, including thrombolysis and percutaneous coronary intervention, has enabled a growing proportion of patients with acute myocardial ischemia to receive timely treatment [2]. However, although reperfusion can salvage ischemic myocardial tissue, it may also trigger additional tissue injury under certain conditions, a phenomenon known as myocardial ischemia-reperfusion injury (MIRI) [1,3]. The pathogenesis of MIRI involves multiple interacting mechanisms, including oxidative stress, calcium overload, inflammatory activation, mitochondrial dysfunction, and programmed cell death, all of which contribute to cardiomyocyte apoptosis and necrosis [4,5]. Secondary injury caused by MIRI reduces the therapeutic benefit of reperfusion and promotes adverse cardiac remodeling and progression to heart failure, ultimately affecting long-term clinical outcomes. Therefore, early identification and dynamic monitoring of MIRI at the tissue level have become critical challenges in basic and clinical cardiovascular research.

Hematoxylin and eosin (H&E)–stained tissue sections remain the diagnostic gold standard for assessing myocardial morphological changes and evaluating cellular injury in both clinical and experimental research. MI lesions identified by H&E staining typically show increased cytoplasmic eosinophilia, loss of cross-striations, and nuclear pyknosis or karyolysis, which indicate coagulative necrosis. These alterations are followed by inflammatory cell infiltration, disruption of myocardial structure, and subsequent fibrosis [6]. Although these histopathological features can be clearly recognized under light microscopy, manual slide evaluation is affected by staining variability, background artifacts, and tissue heterogeneity. These factors reduce analytic efficiency and introduce considerable observer subjectivity [7,8]. Reliance on expert pathological experience further limits the consistency and scalability of H&E slide interpretation, particularly in studies involving large sample sizes, multiple interventions, or multiple time points [9]. Therefore, there is an urgent need for an efficient, objective, and reproducible intelligent method to replace or to assist traditional manual histopathological review (Multimedia Appendix 1).

Artificial intelligence (AI), particularly deep learning, has achieved remarkable advances in medical image analysis in recent years. Convolutional neural networks (CNNs) have been widely applied to the automatic recognition of pathological images in diseases such as breast and lung cancer, demonstrating performance that matches or even exceeds that of human experts [10,11]. Recurrent neural networks (RNNs) and long short-term memory networks (LSTMs) have also shown superior capabilities in handling time series and dynamic medical images [12]. Generative adversarial networks (GANs) provide powerful tools for image augmentation, modality conversion, and synthetic data generation, which effectively mitigate the limitations posed by small training cohorts [13].

Recent systematic analyses have emphasized the expanding role of AI in medical image interpretation, and multiple reviews have noted that deep learning shows considerable potential in pathology, imaging, and multimodal data integration [14,15]. However, most current research remains focused on domains such as tumor image recognition or pulmonary computed tomography analysis. Efforts related to the identification of myocardial reperfusion injury based on histological or imaging data remain limited. Comprehensive studies that integrate multiple models and cross-modality evaluation are still lacking [16]. In addition, the interpretability of model outputs and their alignment with underlying biological mechanisms are still inadequate, further constraining the broad clinical translation of AI technologies in myocardial pathological diagnosis.

Transformer-based models have recently achieved strong performance in natural language processing and image segmentation and are increasingly applied to pathological image interpretation. Their global attention mechanisms enable the capture of long-range pixel dependencies, offering a theoretical advantage in analyzing complex tissue architectures and indistinct boundaries in histological images [17,18]. Generative models such as GANs contribute to data augmentation, rare structure reconstruction, and synthetic sample generation, thereby improving model robustness and generalizability [19]. A systematic comparison of major deep learning architectures for the analysis of H&E-stained sections in MIRI has not yet been established. Relevant architectures include CNNs, RNNs, LSTMs, autoencoders, graph convolutional networks (GCNs), variational autoencoders (VAEs), transformers, and GANs. Studies that analyze dynamic histopathological trends across multiple time points and treatment conditions also remain limited [20]. Current research is further constrained by incomplete dataset construction and inadequate quality control of annotation labels.

A detailed review of current AI applications in medical image recognition provides the basis for the analytical framework developed in this study for MIRI. The framework integrates multiple models, time points, and treatment groups. Eight deep learning architectures are systematically evaluated to identify apoptotic and necrotic regions in H&E-stained myocardial tissue images. These architectures include CNNs, RNNs, LSTMs, autoencoders, GCNs, VAEs, transformers, and GANs. Particular emphasis is placed on the capacity of the transformer architecture to capture dynamic tissue alterations and biologically relevant features. GAN-based models are used for data augmentation and image completion to enhance model generalizability. All training data are sourced from publicly available databases and are reannotated by experienced cardiovascular pathologists to ensure label consistency and dataset reliability. Additionally, we construct a time series dataset of pathological sections to quantitatively track myocardial tissue recovery across different treatment strategies at 6, 24, and 72 hours after reperfusion, thereby providing a novel technical pathway for mechanistic studies and therapeutic evaluation.

In summary, this study aimed to apply deep learning as the central analytical approach to evaluate its ability to perform intelligent recognition and dynamic monitoring of histopathological images in myocardial reperfusion injury. An interpretable AI evaluation framework was established by integrating image, temporal, annotation, and intervention information. Innovation is reflected in both model design and application. The integration of the transformer architecture with GAN models enables automated analysis of H&E-stained images and supports quantitative assessment of myocardial injury progression under different therapeutic strategies. The results provide a high-throughput image analysis tool for basic research and offer clinicians a quantitative decision support system for treatment planning and evaluation. Furthermore, the proposed framework demonstrates strong scalability and can be extended to histopathological analyses of other tissue injury conditions (eg, cerebral infarction, hepatic injury, and pulmonary fibrosis). The approach establishes both the technical and conceptual foundation for developing multimodal, multiscale, and interpretable medical AI platforms with scientific and clinical relevance.

## Methods

### Study Subjects and Sample Acquisition

All myocardial H&E-stained histological images used in this study were obtained from publicly accessible medical image repositories, primarily the BioImage Archive (dataset accession: S-EPMC9426742) and a dataset related to MI and MIRI models released by researchers on Zenodo [21]. The combined dataset comprised a total of 1280 whole-slide images (WSIs) of myocardial HE-stained sections, including 640 human-derived samples (50%) and 640 animal model samples (50%), mainly from Sprague-Dawley rats and C57BL/6 mice. All WSIs were acquired through standardized digital slide scanning procedures and were subsequently processed using a unified tissue detection and image tiling pipeline to generate image patches for model training. Detailed scanning parameters and tiling strategies are described in the subsection “Image Types, Scanning, and Tiling Strategy” below.

Both human myocardial samples and sections from classical murine MIRI models were included in this study based on the well-established evidence that MIRI exhibits highly consistent histopathological end points across species. Previous studies have demonstrated that key pathological features induced by MIRI, such as coagulative necrosis, apoptosis-associated nuclear alterations, and inflammatory cell infiltration, show strong morphological comparability between humans and commonly used rodent models. Moreover, pathological diagnosis of these lesions relies primarily on histological architecture rather than species-specific markers. Accordingly, the inclusion of animal model sections helps to broaden the diversity of pathological morphologies and enhances the robustness of the model to variations in staining protocols, scanning conditions, and tissue heterogeneity. To mitigate potential domain shift arising from cross-species data integration, stratified control was applied during dataset partitioning with respect to species, intervention groups, and time points, and uniform color normalization and quality control procedures were implemented across all samples. It should be emphasized that clinical interpretation and conclusions in this study are primarily based on observations from human-derived samples, while animal samples are mainly used to support model learning of generalizable pathological phenotypes of myocardial reperfusion injury and to evaluate its generalization capability.

All images originated from deidentified public datasets and required no additional ethical approval. No human or animal experiments were performed in this study. Images were included based on the following criteria: (1) H&E-stained sections from clinically confirmed MIRI cases or validated animal models; (2) high image quality with consistent staining and minimal artifacts; (3) presence of typical morphological indicators of myocardial apoptosis or necrosis; (4) availability of multiple reperfusion time points, including 6, 24, and 72 hours; (5) availability of intervention information, including antioxidant, beta-blocker, calcium channel blocker, and control groups; and (6) presence of lesion annotations or descriptive pathological documentation in the original dataset.

To ensure reproducibility and balanced representation, data partitioning was performed at the patient or animal subject level and divided into training, validation, and test subsets in an 8:1:1 ratio. Images from the same subject appeared in only one subset. Stratified sampling across intervention groups and reperfusion time points maintained proportional distributions across subsets.

In addition, the study team invited 2 senior cardiovascular pathology experts, each with more than 15 years of professional experience, to perform secondary validation and fine-grained annotation of key regions on a subset of samples. These annotations were used to calibrate the original labels and improve overall labeling consistency. In cases of disagreement between the 2 experts, arbitration was conducted by a third senior pathologist. The mean overall interannotator agreement, measured by Cohen κ, reached 0.92 (SD 0.03). To clearly present the composition of the dataset, Multimedia Appendix 2 summarizes the category distributions across different treatment groups, time points, and pathological states. According to the expert annotations, the distribution of tissue states was normal:apoptosis:necrosis of 2.1:1.0:1.3, indicating a mild class imbalance. During model training, class weighting and a focal loss–based weighting strategy were applied to mitigate potential bias introduced by this imbalance.

### Image Types, Scanning, and Tiling Strategy

All samples were obtained from the publicly accessible MIRI-Histo v1 database (BioImage Archive, S-EPMC9426742; Zenodo[34], which contains H&E-stained myocardial tissue sections from both human subjects and animal models. A total of 1280 WSIs were included in this study, comprising 640 human-derived samples and 640 animal model samples, primarily from Sprague-Dawley rats and C57BL/6 mice. Human myocardial sections were digitized using an Aperio AT2 scanner at 40× magnification, corresponding to a spatial resolution of 0.25 μm per pixel, whereas animal tissue sections were scanned using a Hamamatsu NanoZoomer S360 scanner at 20× magnification, with a spatial resolution of 0.50 μm per pixel. All images were stored in TIFF or SVS formats and underwent rigorous quality control procedures. Only slides with intact tissue architecture, homogeneous staining, and no evident scanning artifacts were retained for subsequent analysis.

To standardize model inputs and improve training efficiency, a unified tissue region detection and image tiling pipeline was applied to each WSI. First, tissue regions were identified using Otsu thresholding combined with morphological operations to remove background areas. Fragmented regions with an area smaller than 10,000 μm² were excluded. Subsequently, a sliding window strategy was used within the valid tissue regions to generate image tiles, with a tile size of 512×512 pixels and a stride of 256 pixels. Tiles with a tissue occupancy of less than 40% were discarded, retaining only regions with high tissue density. After this procedure, each WSI yielded an average of approximately 50.1 (SD 4.8) valid image tiles.

To further ensure data quality, all image tiles underwent additional screening for sharpness and staining consistency. Image sharpness was assessed using the variance of the Laplacian method, while brightness and chromaticity histograms were used to exclude overexposed images or tiles with abnormal staining. Ultimately, approximately 62,000 high-quality image tiles were obtained. These tiles were partitioned at the subject level into a training set (approximately 49,600 tiles, 80%), a validation set (approximately 6200 tiles, 10%), and a test set (approximately 6200 tiles, 10%), ensuring that images from the same subject appeared in only one subset.

### Image Preprocessing and Data Augmentation

Prior to model training, all image tiles underwent a unified color normalization and preprocessing pipeline. The Macenko algorithm was applied to normalize the color distribution of H&E-stained images, thereby reducing batch effects introduced by variations in experimental conditions and scanning devices. All images were preserved in 3-channel RGB format and processed within the sRGB color space.

To enhance the robustness of the model to variations in tissue morphology and image acquisition conditions, data augmentation strategies were applied exclusively to the training set. These included random rotations (+20° or −20°), horizontal and vertical flipping, brightness adjustments (+10% or −10%), affine transformations, and mild Gaussian blurring. These augmentations were used only during the training phase and were not applied to the validation or test sets.

All image processing procedures were implemented in a Python 3.9 (Python Software Foundation) environment, primarily using OpenCV (OpenCV.org), NumPy (NumFOCUS), Pillow (Pillow Contributors), SimpleITK (Insight Software Consortium), and Albumentations (Albumentations Team) image processing libraries.

### Filtering Validation and Weakly Supervised Label Generation

An identical image filtering workflow was applied across all experimental groups and classification tasks to avoid preprocessing-related bias. Before input into the transformer architecture, each image underwent standardized color normalization, smoothing, and edge enhancement. Gaussian filtering (σ=1.2) was used to reduce scanner noise, and Laplacian operators were applied to sharpen local tissue boundaries. Validation on an independent dataset yielded a mean structural similarity index (SSIM) of 0.982 (SD 0.007) and a mean edge preservation index of 0.963 (SD 0.011) between preprocessed and original images, indicating enhanced textural contrast without significant alterations in cellular boundaries or nuclear density (*P*>.05). Transformer models trained on filtered and unfiltered data showed comparable validation performance: area under the curve (AUC; 0.979 vs 0.982) and *F*_1_-score (0.957 vs 0.958), confirming the neutrality of the filtering procedures.

Several datasets contained expert-annotated lesion regions, from which region of interest (ROI) masks were extracted and spatially aligned using a hybrid registration pipeline based on OpenCV and SimpleITK, consisting of rigid, affine, and B-spline transformations. For datasets lacking manual annotations, a weakly supervised method based on clustering-constrained attention multiple-instance learning (CLAM) was implemented in PyTorch (version 1.13) to generate pseudo-ROIs. Through attention-based learning, the model identified high-contribution regions at the slide level, which were designated as pseudo-ROIs. These pseudo-ROIs were subsequently integrated with expert-annotated samples during feature pretraining and fine-tuning to enhance label consistency and improve model robustness.

### Label Sources and Quality Control

The labeling framework integrated both strong and weak supervision. Pixel-level strong supervision was applied to human samples (n=640), and slide-level weak supervision was used for animal samples (n=640). All annotations originated from the metadata provided in the public databases and were further validated by the research team. The original annotation files contained polygon masks delineating apoptotic regions, necrotic areas, and normal myocardium, generated by expert pathologists at the time of dataset submission and stored in standardized JSON and XML formats.

To ensure label accuracy and consistency, 3 senior cardiovascular pathologists with ≥10 years of diagnostic experience independently reevaluated all slides. A double-blind and randomized review process was conducted in which each expert reinterpreted the slides and confirmed or refined regional annotations based on established histopathological criteria, including apoptosis, necrosis, inflammatory infiltration, and fibrosis. When all reviewers agreed, the annotation was accepted. In cases of disagreement, a consensus was reached by majority vote after adjudication by the third pathologist. Following verification, a total of 12,460 high confidence annotated regions (defined as ≥80% expert agreement) were obtained, corresponding to 62,000 image tiles.

Annotation reliability was assessed using the Cohen κ and Fleiss κ statistics. Pixel-level annotations demonstrated a mean κ of 0.92 (95% CI 0.90‐0.94), while slide-level weak supervision yielded κ of 0.87 (95% CI 0.84‐0.89), indicating excellent interrater agreement. All annotation files underwent logical integrity and spatial consistency checks, including mask-boundary smoothing, hole filling, and area normalization implemented in OpenCV, ensuring cross-format and cross-resolution compatibility.

For weakly supervised samples, a multiple-instance learning (MIL) strategy was used, with global injury scores (0‐1) assigned at the slide level as supervisory labels during model training. The final labeling schema consisted of 3 primary classes (ie, normal myocardium, apoptotic region, and necrotic region) and 2 auxiliary classes (ie, inflammatory infiltration and fibrosis, used only for interpretability analysis and heatmap validation).

### Dataset Splitting and Leakage Prevention

To ensure training independence and reproducibility, all data were divided into training, validation, and independent test sets at an 8:1:1 ratio. Splitting was performed at the subject level (patient or animal level) rather than the patch level to prevent images from the same specimen from appearing in different subsets. The final allocation included 1024 WSIs in the training set (≈49,600 patches), 128 in the validation set (≈6200 patches), and 128 in the test set (≈6200 patches). Each subset was distributionally balanced across species (human or animal), treatment groups (antioxidant, β-blockers, calcium channel blockers, and control groups), and reperfusion time points (6, 24, and 72 h) to ensure comparability in downstream statistical analyses. All splitting procedures were performed using a fixed random seed (seed=42) and repeated 5 times to minimize stochastic bias, with results reported as the average outcome.

Multiple safeguards were used to prevent data leakage during model training and validation. Subject-level splitting ensured that all WSIs and derived patches from a given individual were restricted to a single subset. Temporal independence was maintained by assigning samples from different reperfusion time points (6, 24, and 72 h) independently within each treatment group. Data augmentation procedures, including rotation, flipping, brightness adjustment, blurring, and affine transformation, were applied only to the training set; the validation and test sets remained unchanged. File trace integrity was ensured by assigning each WSI a unique identifier during indexing, and SHA-256 hash verification confirmed that no file or patch appeared in more than one subset. Five-fold cross-validation was additionally performed to evaluate model robustness, and model performance was further validated using an independent external dataset.

The independent test set consisted of 64 human WSIs and 64 animal WSIs, covering all 4 treatment interventions and 3 reperfusion time points. This dataset was used both for quantitative performance evaluation and comparison with expert pathological assessments. All labels in the test set remained completely hidden during model development and tuning and were only accessed after model freezing for inference and final performance assessment, ensuring objectivity and fairness.

### Model Architecture and Training Protocol

#### Overview

All models were developed and optimized within a unified experimental framework to ensure reproducibility. The primary architecture was a ViT-Base/16 model (patch size=16, hidden dimension=768, 12 heads/blocks), using RGB image patches resized from 512×512 to 224×224 pixels as input. Benchmark models included Swin-T, ResNet-50, RNNs or LSTMs, GCNs, autoencoder, VAE, and a GAN-based module used exclusively for synthetic augmentation rather than final classification. Leakage prevention at the slice and subject levels, as well as batch and staining stratification, followed the procedures described in the sections “Label Sources and Quality Control” and “Dataset Splitting and Leakage Prevention.”

#### Sampling Strategy

Each WSI was tiled into 512×512 patches (stride=256) based on tissue masks. Class-balanced sampling (positive:negative=1:1) was applied during training, while validation and test sets followed the natural class distribution. To limit batch variance, no more than 120 patches were randomly sampled from each WSI during each training iteration.

#### Color Processing and Augmentation

Macenko stain normalization was performed prior to modeling. Data augmentation included random rotation (+20° or −20°), horizontal or vertical flipping, ColorJitter (brightness/contrast/saturation/hue=0.2/0.2/0.2/0.05), Gaussian blur (σ=1.0), and elastic deformation (α=15; σ=4), each applied with a probability of .5. Validation and test sets underwent only resizing and center cropping.

#### Optimization and Scheduling

Models were trained using AdamW (learning rate=3×10^−4^, betas=0.9/0.999, weight decay=0.05) with CosineAnnealingLR (T_max=100, min_lr=1×10^–6^). Training parameters included batch size=32, a maximum of 100 epochs, and early stopping based on validation AUC (patience=12, ΔAUC ≥1×10^−3^). Automatic mixed precision (FP16) and gradient clipping (max-norm=1.0) were enabled.

#### Loss Functions and Weighting

Multiclass cross-entropy served as the primary loss function. Class-balanced weighting based on effective sample number was applied when class imbalance exceeded 1.5:1. For weakly supervised branches, Focal loss (γ=2.0; α=.25) was used for pseudo-ROI refinement. Segmentation tasks used a composite Dice+BCE objective (λ_Dice=0.7; λ_BCE=0.3).

#### Label Definitions

According to histopathological criteria, each patch was categorized into noninjured, apoptotic, or necrotic myocardium. Reperfusion time points and treatment groups were used only for stratified or subgroup analyses and were not included as supervised labels.

#### Randomization and Hardware

Deterministic execution was enforced using fixed random seeds {2023, 2024, 2025, 1337, 3407}. Five independent runs were conducted, and results are reported as mean (SD). All training procedures were performed on a single NVIDIA A100 (40 GB) GPU with 256 GB system memory.

#### Reproducibility Materials

Complete YAML configurations, training scripts, and data inventories, including WSI-to-patch mapping and train/val/test lists, are provided in Multimedia Appendix 3.

### Model Selection and Construction

Eight representative deep learning architectures were constructed and compared to evaluate their performance in classifying and recognizing H&E-stained myocardial tissue images following MIRI. The evaluated models included CNNs, RNNs, LSTMs, autoencoders, GANs, GCNs, transformers, and VAEs (Multimedia Appendix 4). CNNs, autoencoders, and GCNs were used mainly for feature extraction and region-level classification in static images. RNNs and LSTMs were designed to process time series labels or sequential image data. Transformers, equipped with global attention mechanisms, demonstrated the ability to model both image and sequential features and offered advantages in medical image interpretation.

Given the relatively limited dataset size, a data augmentation module based on the Deep Convolutional Generative Adversarial Network (DCGAN) framework [22] was constructed to generate high-quality synthetic myocardial H&E images. The GAN architecture consisted of a generator that learned underlying textural patterns and a discriminator that evaluated the authenticity of generated images. The “classification accuracy” reported in Multimedia Appendix 4 reflected only the discriminator’s ability to distinguish real from synthetic samples and served as an indicator of generator convergence. GAN training was restricted to the training set to maintain dataset independence and prevent leakage. After quality filtering, a total of 6250 of 49,600 (12.6%) images in the training set were augmented (synthetically generated). These augmented images were used exclusively during the model training phase to enhance data diversity and improve generalization performance, and they were not included in the validation or test set performance evaluation.

All models were implemented within a unified PyTorch framework, initialized through transfer learning, and optimized using architecture-specific optimizers and learning rate schedulers. Input consisted of preprocessed H&E tiles, and outputs included categorical predictions of myocardial status (normal, apoptotic, and necrotic) or regional annotation maps. Multiple loss functions, including cross-entropy, Dice coefficient, and Focal loss, were applied during training to address class imbalance and subtle histological boundaries. Model performance was evaluated using cross-validation and an independent external test set, assessing accuracy, sensitivity, specificity, robustness, and task-specific metrics for classification, localization, and segmentation tasks. These evaluations enabled the identification of the most suitable architecture for H&E-based myocardial tissue analysis.

### GAN-Based Augmentation Strategy and Leakage Prevention

A StyleGAN2-ADA-based generative adversarial network was incorporated during data augmentation to increase sample diversity and improve model generalization. The generator operated at 512×512 resolution with a 512-dimensional latent space, an 8-layer mapping network, and R1 regularization. The discriminator used adaptive discriminator augmentation to reduce domain shift caused by limited data. GAN training was performed exclusively on real tiles from the training set, following subject-level and slide-level partitioning. The Adam optimizer (β₁=.0; β₂=.99) with an initial learning rate of 2×10^−3^ and exponential decay scheduling was applied. Training continued until convergence criteria were met, corresponding to ~1.2 million real-sample views (kimg ≈ 1200), and stopped early when Fréchet Inception Distance or inception score (IS) stabilization indicated discriminator equilibrium.

During classification and segmentation model training, synthetic GAN-generated samples contributed no more than 10% of input data (real:synthetic ≈9:1). A curriculum mixing strategy was applied, with no synthetic samples during the first 5 epochs, 5% synthetic inclusion during epochs 6 to 10, and 10% thereafter. Synthetic images were excluded from validation and test sets. Leakage prevention followed a strict “split-before-generate” protocol in which dataset partitioning was completed before GAN training, and the generator accessed only the training subset. Real and synthetic samples were stored in separate directories with a syn_ filename prefix, and the dataloader automatically filtered out syn_ files in nontraining modes. Quality control for synthetic images included structural similarity filtering (SSIM ≥0.90), IS thresholds, and near-duplicate detection using perceptual hashing (pHash ≥12) and LPIPS (≥0.20), ensuring removal of synthetic samples that closely resembled real images.

Ablation experiments demonstrated small but consistent improvements with GAN augmentation. For the transformer classifier, the test accuracy increased from 0.942 to 0.944, the AUC from 0.982 to 0.983, and the *F*_1_-score from 0.958 to 0.959. For Transformer-UNet, Dice coefficients for necrosis and apoptosis improved from 0.847 to 0.851 and 0.821 to 0.824, respectively, without statistically significant differences. These results indicate that GAN augmentation slightly improves robustness while preserving evaluation integrity and avoiding synthetic content during validation or testing.

### Evaluation and Validation of GAN-Generated Samples

Quantitative and qualitative assessments were performed to evaluate the data augmentation utility and visual fidelity of synthetic H&E images generated using the DCGAN framework. IS and SSIM were first computed, yielding mean IS of 2.81 (SD 0.17) and mean SSIM of 0.936 (SD 0.012). These results indicated that the synthetic images closely matched real tissue samples in morphological texture and staining distribution. In addition, 100 synthetic and 100 real patches were randomly selected for blinded review by 2 independent pathology experts. Expert identification accuracy was 47.3%, approximating random guessing and confirming the visual realism of the generated images.

The impact of GAN-based augmentation on model performance was further evaluated by training transformer models under identical conditions, with and without synthetic patches. Incorporation of GAN-generated samples increased the validation AUC from 0.978 to 0.982 and improved the *F*_1_-score from 0.956 to 0.958, although neither difference reached statistical significance (*P*>.05). These findings suggest that GAN augmentation provides stable and modest gains in generalization performance without affecting core model conclusions. Overall, the GAN component supports the feasibility of synthetic H&E images as a complementary data expansion strategy and contributes supplementary benefits while not determining the primary analytical outcomes of the study.

### Deep Learning Model Architecture and Implementation Details

Eight mainstream deep learning architectures were implemented within a unified PyTorch (version 1.13) framework to ensure architectural transparency and methodological reproducibility. The evaluated models included CNN, RNN, LSTM, autoencoder, GAN, GCN, VAE, and transformer. All architectures were constructed according to their original seminal publications [22-29] and adapted for the MIRI histopathology classification task using 224×224 RGB H&E image tiles. Standardized, preprocessed patches served as model inputs, and outputs corresponded to 3 tissue categories: normal myocardium, apoptosis, and necrosis. The CNN architecture used a 5-layer convolutional backbone with ReLU activation and max pooling, followed by fully connected layers (512-128-3) and dropout (0.4) to reduce overfitting. RNN and LSTM models were designed for sequential tile modeling, with the LSTM implemented as a bidirectional 2-layer network (hidden size=128 and dropout=0.3). Autoencoders and VAEs performed feature compression and reconstruction with latent dimensions of 128 and 64, respectively; VAE training incorporated a KL divergence regularization term (weight=0.005). The GAN module followed the DCGAN architecture and was applied exclusively for synthetic data augmentation. The GCN treated tile-level features as graph nodes, modeling spatial adjacency to capture microstructural relationships. The core transformer model was built upon the Vision Transformer (ViT-Base) backbone (12 self-attention heads, embedding dimension=768, patch size=16×16) and integrated the CLAM framework to support attention-based weakly supervised MIL, enabling slide-level classification and pseudo-ROI generation. Model-specific loss functions and hyperparameters were selected according to architectural characteristics. Cross-entropy loss was applied to CNN, RNN, LSTM, and GCN models, with additional L2 regularization used for LSTM. Combined mean squared error and SSIM losses were used for autoencoder and VAE models, with KL divergence included for VAE. GAN training used adversarial and discriminator cross-entropy objectives. Class-weighted cross-entropy was adopted for transformer models to address class imbalance. Optimization was performed using Adam or AdamW (learning rate=1×10^−4^; β_1_=0.9; β_2_=0.999), while the transformer used a 3×10^−5^ learning rate with cosine annealing scheduling. Batch sizes ranged from 8 to 64 depending on model complexity. Early stopping (patience=10) was applied to prevent overfitting, and fixed random seeds ensured reproducibility. All models were executed under identical hardware and software environments using publicly available codebases to maintain comparability across architectures.

### Model Training and Evaluation

All deep learning models were trained and evaluated using the preprocessed H&E-stained myocardial tissue images, following a unified data partitioning strategy and standardized training pipeline to ensure comparability across architectures. The dataset was divided into training, validation, and test sets in an 8:1:1 ratio, with balanced representation of cardiomyocyte states (normal, apoptotic, and necrotic) in each subset. Model training was conducted in an NVIDIA GPU–accelerated environment using Adam or RMSProp optimizers. Adaptive learning rates and batch sizes were configured according to the requirements of each model. Early stopping and learning rate scheduling were applied to prevent overfitting during optimization.

Model performance was assessed using accuracy, sensitivity, specificity, precision, *F*_1_-score, the AUC, and Dice coefficient to evaluate both classification and segmentation tasks. For multiclass classification, all metrics were calculated using the weighted macro-average method to ensure balanced contributions across classes. In segmentation tasks, particular emphasis was placed on the identification of boundary regions and small lesions. Dice loss and focal loss were used to improve sensitivity to target structures.

Transfer validation was conducted using multiple independent external datasets to further evaluate model generalizability. Model predictions were compared with expert-annotated labels, and agreement between the AI model and clinical experts was quantified using the Cohen κ coefficient. For images generated by GANs, objective assessments of image quality were performed using the SSIM and IS to validate the effectiveness of GANs in data augmentation. On the basis of combined performance across validation and test sets, the suitability of all 8 models for myocardial injury detection was assessed. Special attention was given to the best-performing transformer-based model, particularly its stability and advantages across multiple time points, injury categories, and regions characterized by blurred pathological boundaries.

### Comparative Analysis of Therapeutic Strategies

MIRI samples with known treatment backgrounds were selected from the publicly available BioImage Archive database. The dataset included several representative intervention strategies: antioxidant therapy, calcium channel blockade, β-adrenergic receptor inhibition, and an untreated control group. Each group contained H&E-stained tissue sections corresponding to multiple reperfusion time points (eg, 6, 24, and 72 h), providing the temporal coverage required for longitudinal evaluation. Using the best-performing transformer model, an automated recognition pipeline was constructed to extract key pathological indicators at each time point, including the proportion of apoptotic cells, necrotic area ratio, and tissue integrity scores. These indicators served as primary metrics for evaluating therapeutic efficacy.

During AI-based analysis, the model received preprocessed H&E-stained images and learned to identify characteristic morphological patterns of injury, including nuclear pyknosis, cytoplasmic homogenization, myofiber disruption, and blurred cellular boundaries. Multidimensional quantitative outputs were generated for each sample. Cross-validation and external test sets were used to evaluate model generalizability across treatment groups, ensuring objectivity and reproducibility. Conventional pathological scoring systems were incorporated as reference standards to compare and validate model predictions.

By establishing a closed-loop workflow encompassing “treatment strategy → image input → AI analysis → extraction of injury metrics,” this study enables an intelligent, quantitative assessment of different therapeutic interventions. The proposed framework lays a methodological foundation for future treatment optimization and mechanistic exploration.

### Temporal Evaluation of Posttreatment Effects

To assess the dynamic therapeutic effects of different interventions during the recovery phase of MIRI, we performed a longitudinal analysis using the best-performing transformer model. All treatment groups were analyzed using H&E-stained myocardial tissue sections obtained at multiple reperfusion time points (eg, 6, 24, and 72 h). The model automatically identified key pathological features, including apoptotic cells, necrotic regions, and structural disorganization, and quantified them into interpretable metrics, such as necrosis area ratio, apoptosis proportion, and tissue integrity score. These outputs were used to construct temporal progression curves of myocardial injury.

We analyzed the temporal dynamics of tissue recovery for the intervention group with the most significant treatment effect to explore the time window in which early intervention alleviates pathological changes. The time series results of the other treatment groups were also identified synchronously by the model as comparative data, further validating the broad applicability of AI-based recognition in dynamic efficacy evaluation.

All time series images were strictly aligned during training to ensure consistency in image regions, tissue layers, and lesion areas. The model’s output results were used to generate trend curves through linear fitting or locally estimated scatterplot smoothing, and the statistical significance of changes across different time points was assessed using ANOVA and repeated measures *t* tests. These analyses provide image-based support for understanding the timeliness and reversibility of treatment responses following reperfusion injury.

### Statistical Analysis

All statistical analyses were performed using R software (version 4.2.2) and SPSS (version 26.0; IBM Corp). For continuous variables derived from model outputs, including the proportion of apoptotic regions, necrotic area percentage, and tissue structure scores, descriptive statistics were calculated as mean (SD) or median as appropriate. Comparisons among multiple groups were conducted using one-way ANOVA. When significant differences were detected, post hoc pairwise comparisons were performed using either the Least Significant Difference test or Tukey’s Honest Significant Difference test. For data with a temporal structure (eg, pretreatment, 6, 24, and 72 h post treatment), repeated measures ANOVA was used to assess the effects of time, treatment, and their interaction on tissue injury parameters. When necessary, paired *t* tests were used for within-group comparisons.

For the evaluation metrics of AI model classification tasks (such as accuracy, sensitivity, specificity, *F*_1_-score, and AUC), the average values from cross-validation were used for summary, along with calculating their 95% CIs. The DeLong test was used to assess performance differences between models for statistical comparison of receiver operating characteristic (ROC) curves. In image segmentation tasks, the Dice coefficient and intersection over union are used to evaluate the consistency between predictions and expert annotations, while Cohen κ coefficient is used to further quantify the correlation between AI and human recognition results.

All statistical tests were 2-tailed unless otherwise specified, and a *P* value of <.05 was considered statistically significant. The results of these analyses were used to support model evaluation, intergroup treatment comparisons, and the assessment of time-dependent changes, ensuring statistical rigor and reproducibility of the study findings.

### Ethical Considerations

All myocardial H&E-stained histopathological images used in this study were obtained exclusively from publicly accessible data repositories, and no new human or animal experiments were conducted. The primary dataset, MIRI-Histo v1, is available through the BioImage Archive (accession: S-EPMC9426742) and the Zenodo data sharing platform [34].

All images were fully deidentified, and the original annotation files (JSON and mask formats) are provided to support reproducibility and secondary analysis. Model training was performed in a Python 3.9 environment using open-source libraries, including PyTorch, OpenCV, NumPy, Pillow, and Albumentations. All source code necessary to reproduce the results will be released publicly upon formal publication.

As this study exclusively used publicly available, deidentified medical imaging datasets, no additional institutional review board or ethics committee approval was required. Data collection and sharing procedures for the original datasets complied with institutional ethics standards and data protection regulations, including the European General Data Protection Regulation and policies of the BioImage Archive. The research team was not involved in any direct acquisition of human or animal specimens, and all data usage adhered to international standards for ethical research and responsible data stewardship.

## Results

### Transformer Model Demonstrates Superior Overall Recognition Performance

A total of 1280 myocardial H&E WSIs and approximately 62,000 high-quality tissue patches were included in the analysis. The dataset was evenly distributed across 4 therapeutic intervention groups (antioxidant, β-blockers, calcium channel blockers, and untreated controls) and 3 reperfusion time points (6, 24, and 72 h), with balanced representation of human and animal samples. This dataset constituted the foundation for training, validation, and testing of all 8 deep learning models.

**Figure 1.**
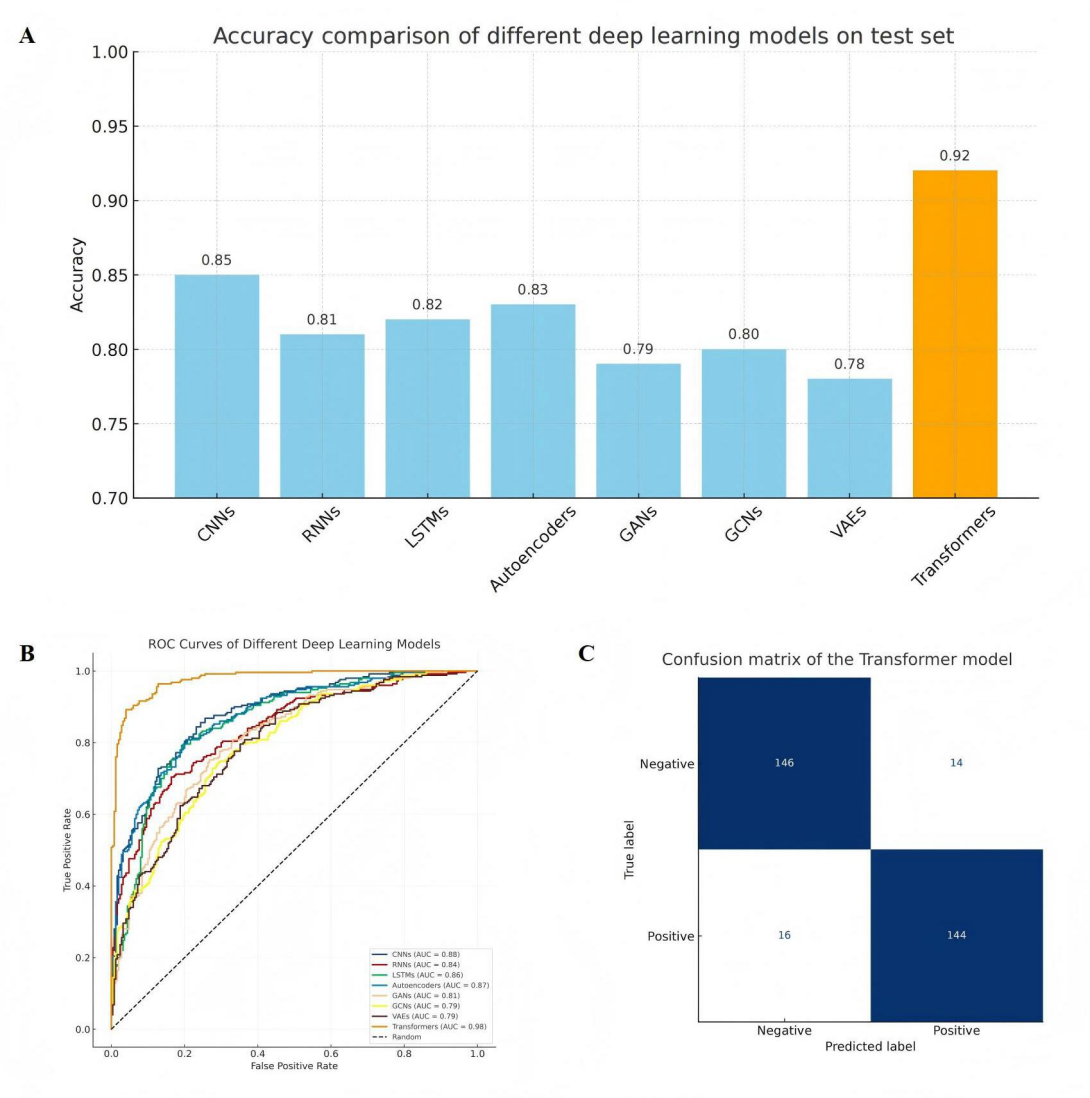
Comparative performance of different deep learning models in the recognition of MIRI on H&E-stained histological images. (A) Classification accuracy of 8 representative deep learning models on the test set. The transformer model outperforms all others in overall accuracy. (B) Receiver operating characteristic (ROC) curves for each model. The transformer model achieves the highest AUC value of 0.963. (C) Confusion matrix of the transformer model, demonstrating the lowest false-positive and false-negative rates among all models, indicating superior classification consistency and boundary recognition capability. CNN: convolutional neural network; GAN: generative adversarial network; GCN: graph convolutional network; LSTM: long short-term memory; RNN: recurrent neural network; VAEs: variational autoencoders.

Eight representative deep learning architectures were implemented to systematically evaluate computational performance in identifying MIRI patterns on H&E-stained histopathology images. The models included CNNs, RNNs, LSTMs, autoencoders, GANs, GCNs, VAEs, and transformers (Figure 1A).

All models were evaluated using the same data split and assessed with 5 complementary metrics: accuracy, precision, recall, *F*_1_-score, and AUC. The transformer model demonstrated the strongest overall performance, achieving a mean cross-validation accuracy of 0.920 (SD 0.011) and a mean final independent test accuracy of 0.942 (SD 0.008), with a mean AUC of 0.982 (SD 0.004), outperforming all comparator architectures (Figure 1B). Confusion matrix analysis further revealed markedly reduced false-positive and false-negative rates (Figure 1C), indicating superior sensitivity, specificity, and robustness in myocardial tissue classification.

### Quantitative Performance Statistics

To ensure reproducibility and enable cross-study comparison, the classification performance of all 8 model architectures was evaluated using an identical dataset split and a unified assessment protocol. Table 1 reports point estimates and 95% CIs for accuracy, AUC, *F*_1_-score, precision, and recall, calculated using nonparametric bootstrapping (n=1000). Consistent with the overall findings, the transformer model achieved the strongest performance across all evaluation metrics. Both the 5-fold cross-validation mean accuracy (0.920) and the independent held-out test accuracy (0.942) are presented to reflect model behavior under different statistical evaluation schemes.

**Table 1.** Classification metrics across models (5-fold CV^a^ mean [95% CI]) and hold-out test^b^.

Model	Accuracy (CV)	AUC^c^ (CV)	*F*_1_-score (CV)	Precision (CV)	Recall (CV)	Accuracy (test)
Transformer	0.920 [0.912‐0.928]	0.963 [0.956‐0.970]	0.958 [0.951‐0.964]	0.955 [0.947‐0.962]	0.962 [0.955‐0.969]	0.942
CNN^d^	0.889 [0.879‐0.899]	0.935 [0.926‐0.944]	0.927 [0.918‐0.936]	0.918 [0.908‐0.928]	0.937 [0.928‐0.946]	0.908
LSTM^e^	0.874 [0.864‐0.885]	0.922 [0.912‐0.933]	0.913 [0.903‐0.924]	0.905 [0.893‐0.916]	0.922 [0.912‐0.933]	0.895
RNN^f^	0.862 [0.851‐0.873]	0.913 [0.902‐0.925]	0.902 [0.890‐0.913]	0.894 [0.881‐0.906]	0.911 [0.899‐0.923]	0.882
Autoencoder	0.848 [0.836‐0.860]	0.902 [0.889‐0.915]	0.889 [0.877‐0.901]	0.882 [0.868‐0.895]	0.897 [0.885‐0.910]	0.871
GCN^g^	0.857 [0.845‐0.869]	0.909 [0.897‐0.921]	0.897 [0.885‐0.909]	0.890 [0.877‐0.903]	0.904 [0.892‐0.916]	0.879
VAE^h^	0.842 [0.830‐0.855]	0.898 [0.885‐0.911]	0.885 [0.872‐0.898]	0.877 [0.863‐0.891]	0.893 [0.880‐0.906]	0.868
GAN^i^	0.801 [0.787‐0.815]	0.861 [0.847‐0.875]	0.846 [0.832‐0.860]	0.839 [0.824‐0.854]	0.854 [0.840‐0.869]	0.829

a CV: cross-validation.

b This table summarizes the classification performance of 8 deep learning models (CNN, RNN, LSTM, Autoencoder, GAN, GCN, VAE, and transformer) on 5-fold cross-validation and the independent test set. Evaluation metrics include accuracy, area under the curve (AUC), *F*_1_-score, precision, and recall, with 95% CIs calculated using 1000 bootstrap iterations. The results show that the transformer model achieves the best performance across all metrics, demonstrating high stability and strong generalization capability.

c AUC: area under the curve.

d CNN: convolutional neural network.

e LSTM: long short-term memory.

f RNN: recurrent neural network.

g GCN: graph convolutional network.

h VAE: variational autoencoder.

i GAN: generative adversarial network.

### Quantitative Evaluation and Comparative Assessment of Classification Performance

**Figure 2.**
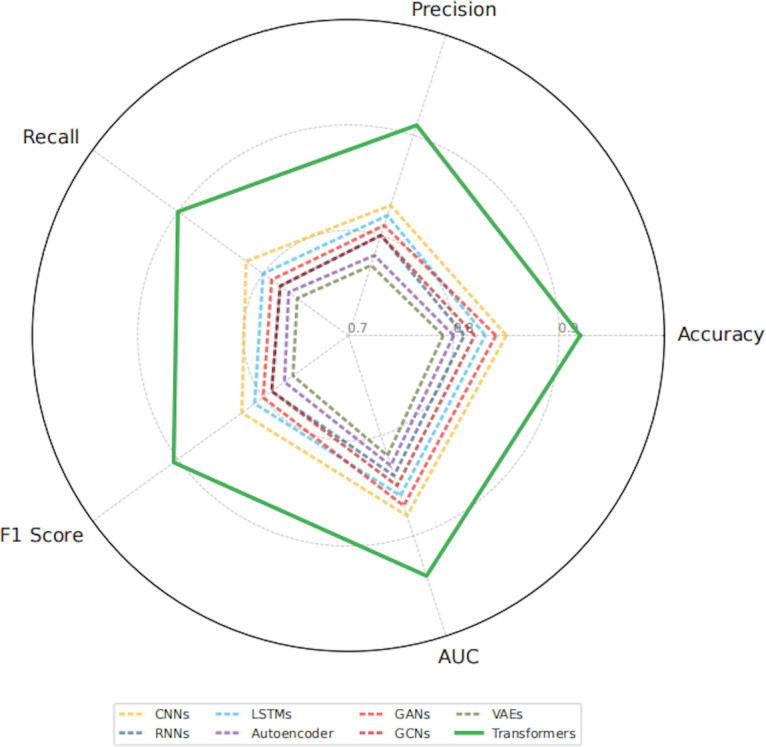
Transformers achieve the best overall performance across 5 key metrics. Radar plot showing the aggregated performance of all deep learning models across 5 evaluation metrics—accuracy, precision, recall, *F*_1_-score, and area under the curve (AUC). The transformer architecture outperforms all other models across every dimension, demonstrating superior discriminatory power and generalizability, particularly for complex tissue structures and highly heterogeneous lesion patterns. CNN: convolutional neural network; GAN: generative adversarial network; GCN: graph convolutional network; LSTM: long short-term memory; RNN: recurrent neural network; VAEs: variational autoencoders.

In the aggregated comparison of classification performance, the transformer model ranked first across all 5 evaluation metrics, including accuracy, *F*_1_-score, AUC, precision, and recall. A mean test set accuracy of 94.2% (*P*<.01) demonstrated significantly superior performance relative to all other architectures (Figure 2). Strong robustness was observed in challenging histopathological conditions, including blurred tissue boundaries, disorganized myocardial structures, and heterogeneous lesion patterns. Supplementary analyses further indicated shorter inference time and faster convergence during training, supporting the feasibility of the transformer architecture for practical deployment.

Although GANs were not used as primary classifiers, they contributed indirectly by supplying high-quality synthetic augmentation samples, thereby enhancing downstream discriminative model stability and generalization capacity. Dual-metric evaluation using the IS and SSIM confirmed that GAN-generated images achieved high diversity and structural fidelity, reinforcing their value as an auxiliary data enhancement strategy.

To further characterize differences in computational efficiency and deployment readiness, additional quantitative indicators were recorded, including training loss fluctuation, the number of epochs required for convergence, and mean per-image inference latency. Detailed values are reported in Multimedia Appendix 5. Overall, the transformer model retained the highest accuracy while exhibiting favorable convergence behavior and strong generalization consistency. Although training loss variability (SD 0.184) was slightly greater than that of lighter architectures, stable validation curves, absence of overfitting, and reasonable inference speed underscore its potential for clinical integration and scalable biomedical imaging applications.

### Segmentation Performance and Consistency Validation

Segmentation performance for necrotic and apoptotic myocardial regions was evaluated using the Dice coefficient and Intersection over Union. Consistent with the classification results, the Transformer-UNet architecture achieved the highest agreement with expert annotations across both mask types (Table 2). All values are reported as mean (SD) on the held-out test set.

**Table 2.** Segmentation performance by lesion type^a^.

Model (segmentation)	Dice (necrosis), mean (SD)	IoU^b^ (necrosis), mean (SD)	Sensitivity (necrosis), mean (SD)	Specificity (necrosis), mean (SD)	Dice (apoptosis), mean (SD)	IoU (apoptosis), mean (SD)	Sensitivity (apoptosis), mean (SD)	Specificity (apoptosis), mean (SD)
Transformer-UNet	0.847 (0.032)	0.740 (0.038)	0.861 (0.035)	0.973 (0.010)	0.821 (0.036)	0.696 (0.041)	0.838 (0.041)	0.968 (0.012)
CNN-UNet	0.812 (0.037)	0.683 (0.043)	0.829 (0.042)	0.965 (0.013)	0.788 (0.041)	0.653 (0.046)	0.808 (0.047)	0.960 (0.014)

a The table presents the main quantitative performance metrics of Transformer-UNet and CNN-UNet in the task of segmenting myocardial ischemia-reperfusion injury regions, including Dice coefficients, IoU, sensitivity, and specificity for necrotic and apoptotic areas. Agreement analysis between model predictions and manual annotations (Cohen κ) is reported separately in the main text.

b IoU: intersection over union.

To further quantify agreement between model predictions and human experts, we computed Cohen κ. The overall κ was 0.86 (95% CI 0.83‐0.89), indicating strong agreement. Stratified analyses across reperfusion time points showed progressively increasing consistency, with mean values of 0.83 (SD 0.02) at 6 hours, 0.86 (SD 0.02) at 24 hours, and 0.88 (SD 0.01) at 72 hours, suggesting that pathological regions became more distinguishable over time. For comparison, interexpert agreement between the 2 board-certified cardiovascular pathologists reached a κ of 0.88 (95% CI 0.85‐0.91), demonstrating high stability of the reference standard. The close similarity between model-expert and expert-expert κ values supports the reliability, interpretability, and clinical applicability of the segmentation model.

### Transformer Model Exhibits Stable Convergence and Strong Generalization During Training

**Figure 3.**
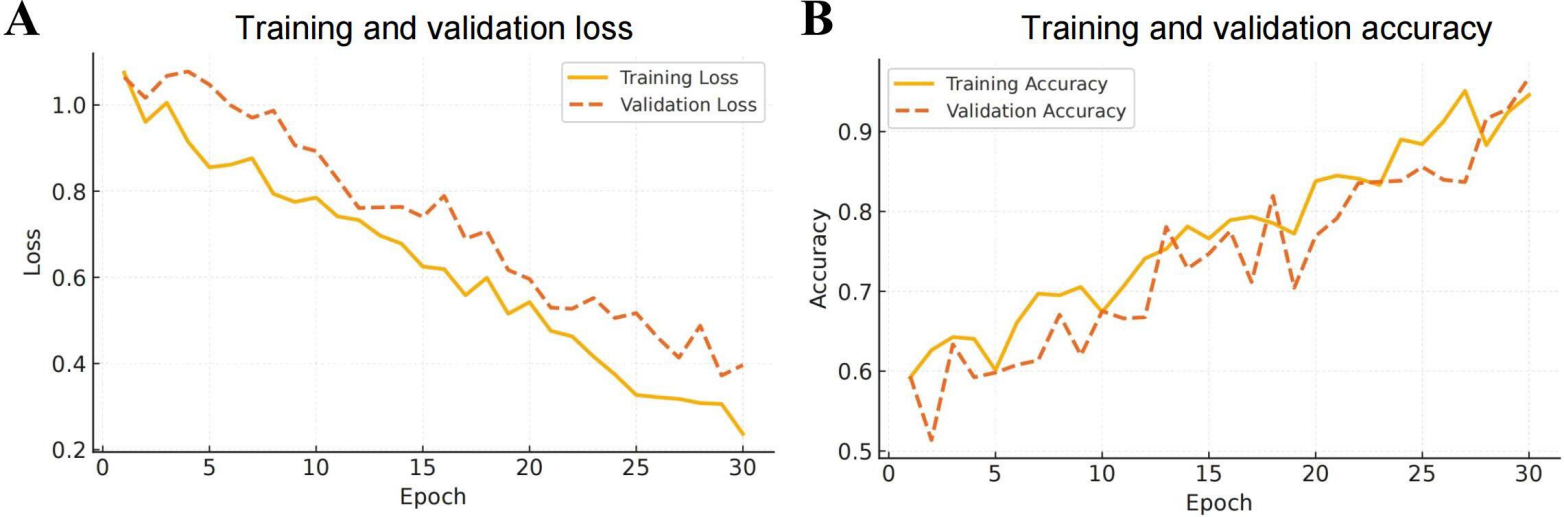
Training dynamics of the transformer model showing loss reduction and accuracy improvement. (A) Loss function curves for the training and validation sets, showing that the model gradually converged during training without oscillation or overfitting. (B) The accuracy of the training and validation sets increased progressively with the number of iterations, indicating that the model had a stable learning process and good generalization ability.

Training process analysis demonstrated that the transformer model displayed a continuous decrease in loss for both the training and validation sets, accompanied by steadily increasing accuracy and no evidence of overfitting (Figure 3A). The learning trajectory remained smooth, and convergence was stable across training epochs, reflecting reliable optimization behavior. Strong generalization performance was observed on the validation set, with consistent accuracy maintained across different dataset partitions. The results indicate that the transformer architecture effectively learns pathological image features and provides robust, transferable performance in myocardial tissue recognition tasks (Figure 3B).

### Comparison of Structural and Functional Pathological Features Across Treatment Groups

After confirming the superior classification performance of the transformer model for myocardial H&E-stained images, the model was applied to automatically identify and quantitatively assess pathological alterations across different treatment groups to evaluate the tissue repair effects of various interventions in MIRI.

**Figure 4.**
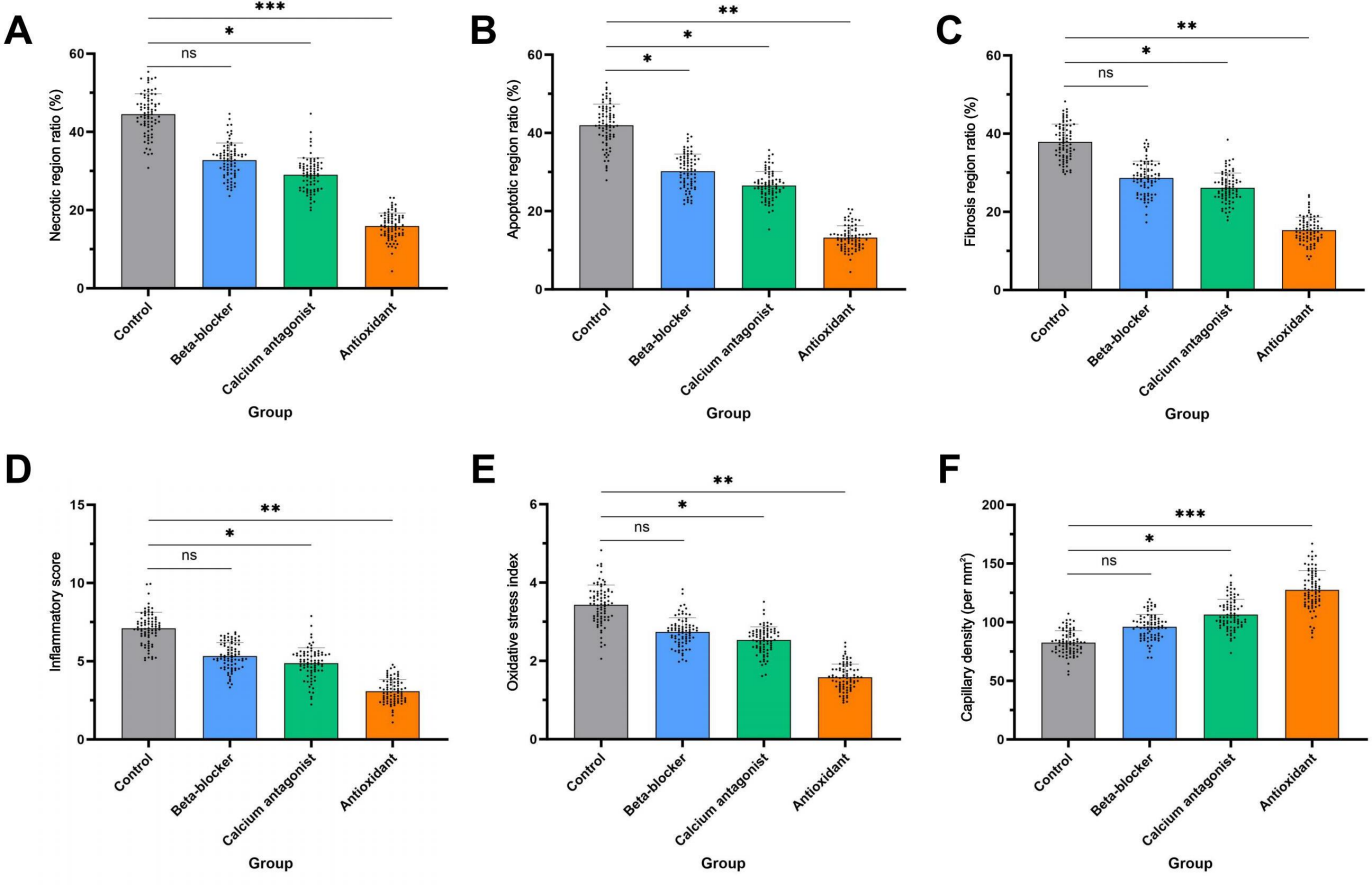
Evaluation of the effects of multiple therapeutic interventions on structural and functional tissue parameters following myocardial ischemia-reperfusion injury. (A-C) Comparisons of necrotic area percentage, apoptotic area percentage, and fibrosis scores among treatment groups indicate that the antioxidant group shows the most significant structural protection. (D-F) Group comparisons of inflammation scores, oxidative stress indices, and capillary density reveal that the antioxidant group performs better in modulating immune responses, providing antioxidant protection, and maintaining microcirculation. Statistical significance was assessed using one-way ANOVA followed by post hoc multiple comparisons. *P<.05; **P<.01; ***P<.001; ns: no significant difference.

For structural damage, the antioxidant-treated group exhibited the most pronounced reduction in necrotic and apoptotic regions, indicating better preservation of membrane integrity and more effective suppression of programmed cell death. The control group showed the highest proportions of necrosis and apoptosis, reflecting the most severe tissue injury. The β-blocker group demonstrated a stronger inhibitory effect on apoptosis, whereas the calcium channel blocker group showed modest antifibrotic potential during structural remodeling (Figures 4A-4C).

For functional pathological markers, the antioxidant group displayed the lowest inflammatory response scores and oxidative stress index values, suggesting a broad protective effect potentially mediated through modulation of immune activity and reduction of free radical accumulation. Increased capillary density scores further indicated improved microvascular integrity, which may have contributed to more stable tissue repair progression (Figures 4D-4F).

### Discriminative Accuracy Performance of the Transformer Model in Myocardial Injury Images Across Multiple Intervention Groups

**Figure 5.**
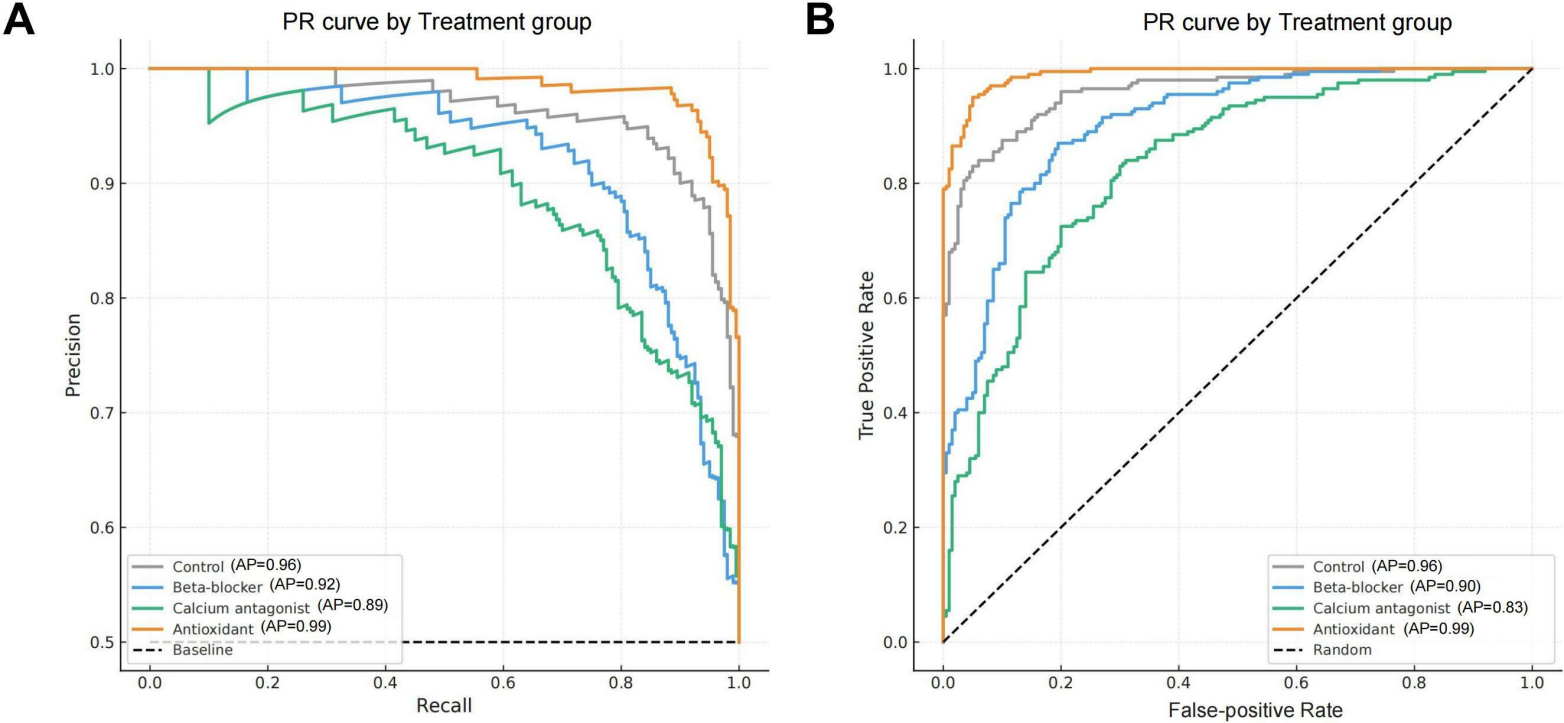
Evaluation of transformer model performance under 4 therapeutic interventions. (A) The precision-recall (PR) curves of the 4 treatment groups demonstrate the model’s classification accuracy under different intervention conditions, with the antioxidant group showing the highest average precision (AP) value, indicating the strongest sensitivity of the model in this group. (B) The corresponding receiver operating characteristic (ROC) curves indicate that the antioxidant group also has the highest area under the curve (AUC), suggesting optimal model discriminative power for this image category. The calcium channel blocker group shows the weakest performance, implying that the complexity of its image features may pose greater challenges for the model.

To further evaluate the transformer model’s image discrimination capability under different treatment interventions, this study plotted precision-recall (PR) curves and ROC curves for 4 treatment groups (antioxidant, β-blockers, calcium channel blockers, and control group) to quantitatively assess the model’s classification accuracy and discriminative power in a complex multigroup setting (Figures 5A and 5B).

The results showed that the Transformer model achieved the strongest performance in the antioxidant group, reaching an average precision and a mean AUC of 0.99 (SD 0.003) and 0.99 (SD 0.004), respectively. These values indicate highly reliable recognition of subtle repair trends and boundary features within antioxidant-treated samples. The control group ranked second, with AP of 0.96 (SD 0.006), and the AUC was also 0.96 (SD 0.005). Strong performance in this group was expected, as the classification task was less challenging due to the consistent and severe tissue damage, which provided clearer morphological cues.

Performance in the β-blocker and calcium channel blocker groups was lower, with mean AUC values of 0.90 (SD 0.011) and 0.83 (SD 0.015), respectively, accompanied by modest PR metrics. Increased morphological heterogeneity and less distinct lesion boundaries in these groups likely contributed to the reduced discriminative accuracy by creating more complex recognition conditions for the model. Overall, the transformer model demonstrated particular strength in processing images with pronounced structural continuity and clear repair dynamics, such as those observed in the antioxidant group, highlighting its advantage in global feature modeling and contextual pattern perception.

### Analysis of Multigroup Image Recognition Stability and Model Discriminative Power Based on Confidence Scores

**Figure 6.**
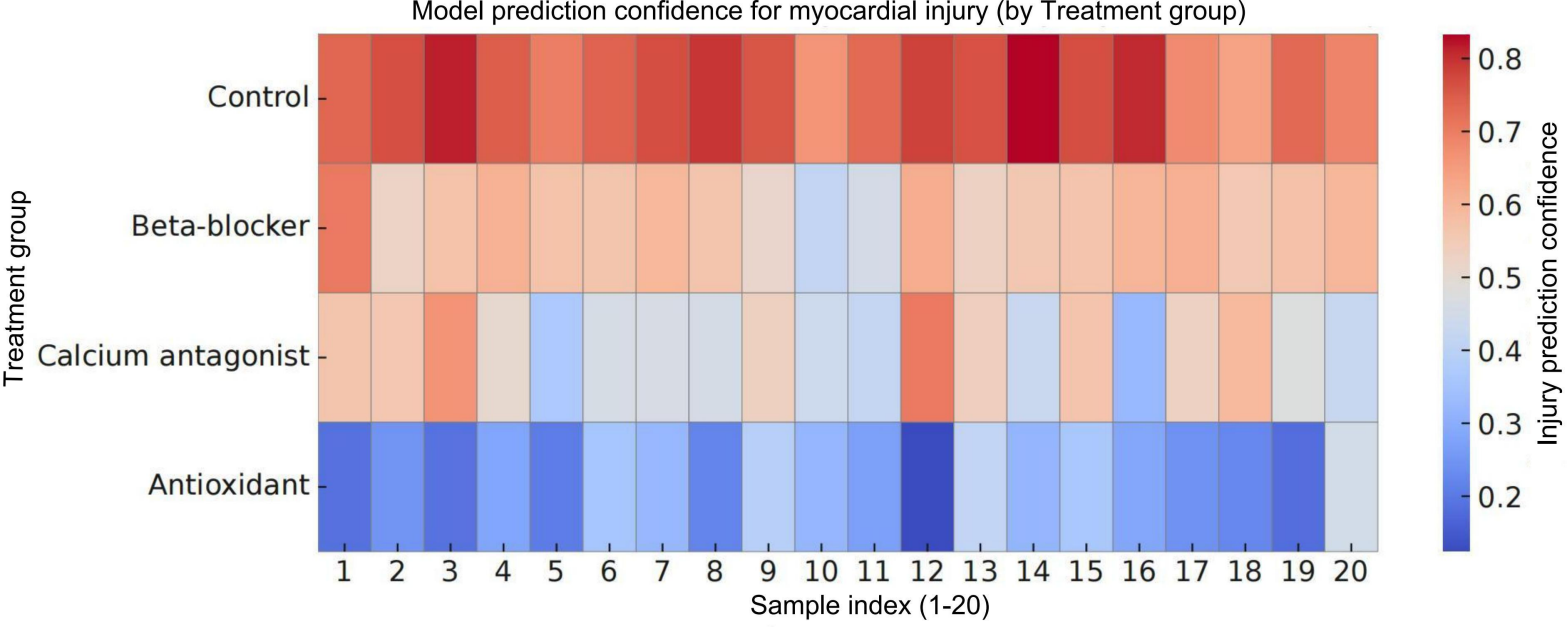
Heatmap of tissue damage prediction confidence by the transformer model across treatment groups. The heatmap illustrates the distribution of damage prediction confidence scores for 20 myocardial hematoxylin and eosin–stained image samples per group under 4 treatment conditions. The color gradient ranges from blue (low confidence) to red (high confidence), indicating the model’s predicted probability of tissue damage. Samples from the control group are predominantly concentrated in high confidence regions (red), while those from the antioxidant group are primarily located in low-confidence areas (blue), reflecting the model’s discriminative sensitivity and stratification stability across different treatment backgrounds.

To further explore the prediction stability and confidence distribution patterns of the transformer model in classifying myocardial injury images under different therapeutic conditions, we generated a 2D heatmap based on model output confidence scores (Figure 6). Each treatment group included 20 samples. The vertical axis represents the treatment categories, and the horizontal axis denotes individual sample identifiers. The color gradient extends from blue to red, corresponding to low and high predicted probabilities for the tissue injury label, respectively.

The results showed that samples from the control group exhibit consistently high confidence in predicted injury status, with the heatmap largely distributed in the red region. This indicates pronounced tissue damage and strong prediction consistency across control samples. In contrast, the antioxidant group predominantly displays low-confidence scores (mostly blue), suggesting that the model identifies milder tissue alterations or a tendency toward repair, with predictions leaning toward “no or mild injury.”

The β-blocker and calcium antagonist groups demonstrated intermediate confidence distributions, characterized by noticeable individual variability and gradual color transitions. These patterns may reflect heterogeneous tissue responses to the respective therapeutic interventions. Overall, the confidence scores showed clear stratification across treatment groups, confirming strong discriminative capability and stable performance of the model in multiclass classification. The confidence-based visualization further supported biological plausibility and enhanced the interpretability of the predictions.

### Temporal Evolution of Myocardial Injury Under Different Treatment Strategies

**Figure 7.**
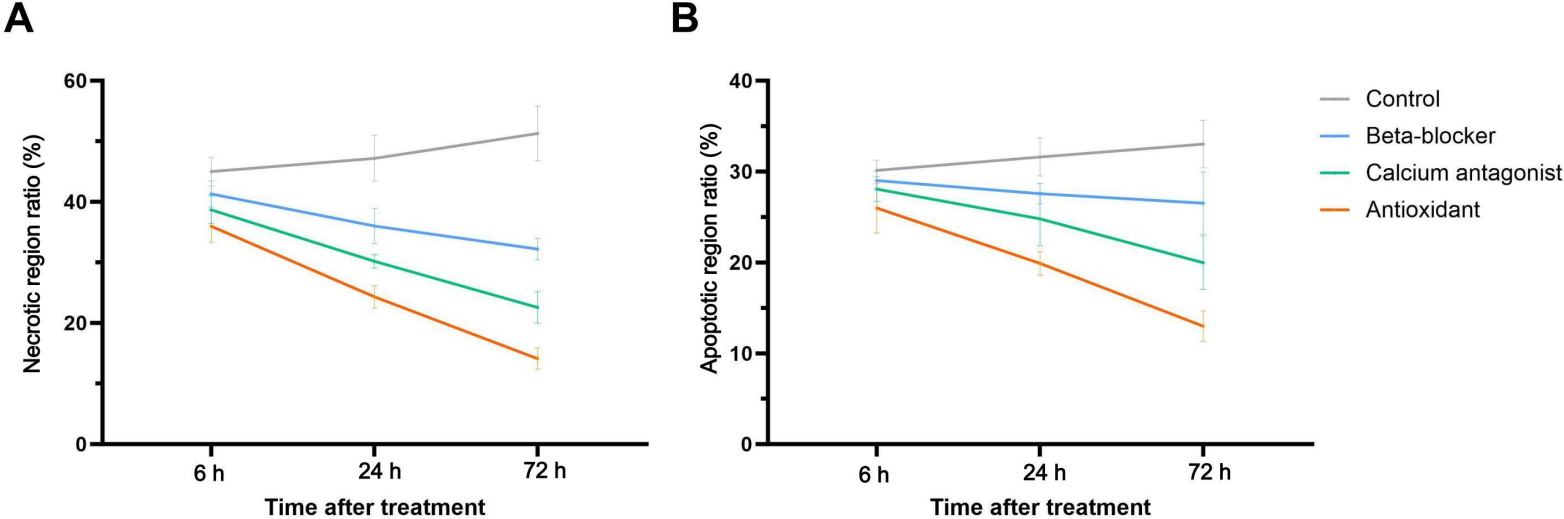
Dynamic trends of key myocardial injury indicators at 3 time points across different treatment groups. (A) The necrotic area ratio over time shows the most substantial reduction in the antioxidant group by 72 h, indicating a strong tissue-protective effect. In contrast, the control group exhibits a continuous upward trend, suggesting progressive damage without intervention. (B) A similar trend is observed for the apoptotic area ratio, with the antioxidant group showing the most significant decline, while other groups demonstrate only mild decreases, and the control group slightly increases. Error bars represent each group’s SD.

To evaluate the discriminative performance of the transformer-based model on time series images, 3 key time points after myocardial reperfusion—6, 24, and 72 hours—were selected. On the basis of the model’s automated identification results, dynamic changes in 2 primary injury-related indicators were analyzed across different treatment groups: the proportion of necrosis-related patches and the proportion of apoptosis-related patches (Figures 7A and 7B). All proportions were calculated as the number of patches classified by the model as injured divided by the total number of patches within the corresponding group at each time point.

The results showed that the antioxidant-treated group exhibited a significant and sustained downward trend in both indicators. Specifically, the proportion of necrosis-related patches decreased from 461 of 1282 (36.0%) at 6 hours to 311 of 1279 (24.3%) at 24 hours, and further declined to 183 of 1295 (14.1%) at 72 hours (Figure 7A). A consistent trend was observed for apoptosis-related patches, with proportions decreasing from 334 of 1285 (26.0%) at 6 hours to 256 of 1286 (19.9%) at 24 hours, and reaching 168 of 1292 (13.0%) at 72 hours (Figure 7B). These findings indicate that antioxidant intervention exerts a pronounced tissue-protective effect as early as the initial phase of reperfusion, and that this protective effect progressively strengthens over the follow-up period.

In contrast, the β-adrenergic blocker group and the calcium channel blocker group showed a certain degree of injury attenuation at the 24-hour time point, but the overall downward trend plateaued after 72 hours. In the β-adrenergic blocker group, the proportion of necrosis-related patches decreased from 524 of 1269 (41.3%) at 6 hours to 458 of 1271 (36.0%) at 24 hours and was 414 of 1286 (32.2%) at 72 hours. The corresponding proportions of apoptosis-related patches were 368 of 1269 (29.0%), 351 of 1273 (27.6%), and 342 of 1289 (26.5%), respectively. A similar pattern was observed in the calcium channel blocker group, where the proportion of necrosis-related patches declined from 489 of 1265 (38.7%) at 6 hours to 384 of 1272 (30.2%) at 24 hours, and further to 292 of 1294 (22.6%) at 72 hours; the corresponding apoptosis-related patch proportions were 356 of 1268 (28.1%), 316 of 1275 (24.8%), and 258 of 1292 (20.0%). These findings suggest that both interventions confer a moderate protective effect during the intermediate phase, but their capacity to promote long-term tissue repair is relatively limited.

In contrast, the control group exhibited a progressive worsening over time in both injury-related indicators. The proportion of necrosis-related patches increased from 587 of 1304 (45.0%) at 6 hours to 620 of 1314 (47.2%) at 24 hours and further rose to 677 of 1320 (51.3%) at 72 hours. Similarly, the proportion of apoptosis-related patches increased from 394 of 1307 (30.1%) to 416 of 1316 (31.6%), reaching 437 of 1323 (33.0%) at 72 hours. These results indicate that, in the absence of therapeutic intervention, myocardial tissue injury continues to progress with reperfusion time, with no clear evidence of spontaneous repair.

Overall, distinct treatment strategies exhibit pronounced time-dependent differences in the histopathological evolution of MIRI. The transformer-based model is able to stably and sensitively capture these dynamic changes at the patch level, supporting its potential utility in mechanistic studies of reperfusion injury and in stage-wise evaluation of therapeutic efficacy.

### Evaluation of the Discriminative Performance of Transformer Models Across Different Epochs

**Figure 8.**
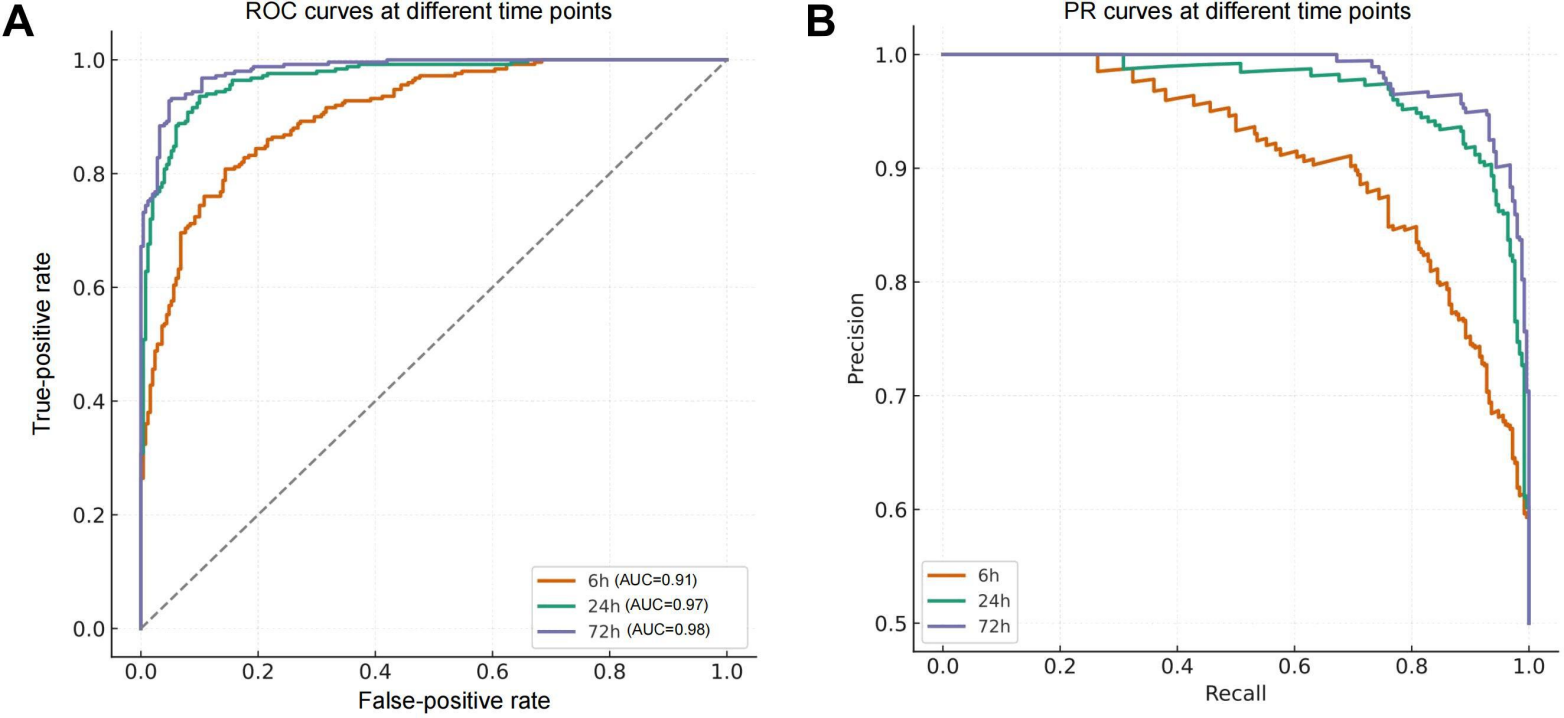
Evaluation of classification performance of the transformer model on myocardial injury images at 3 time points. (A) The receiver operating characteristic (ROC) curve comparison indicates that the model achieves the highest area under the curve (AUC) of 0.98 at 72 h, demonstrating excellent sensitivity to mild late-stage injury. The AUC values at 24 and 6 h are 0.97 and 0.91, respectively, confirming the model’s stable performance in identifying acute-phase lesions. (B) The precision-recall (PR) curve demonstrates that the model achieves the highest balance between precision and recall at 72 h, confirming its generalization capability across time and consistent performance in early- and late-stage recognition.

To validate the transformer model’s classification performance across different postreperfusion stages, we generated ROC and PR curves for 3 key time points: 6 hours, 24 hours, and 72 hours (Figures 8A and 8B). The results demonstrate that the model achieved its strongest performance at 72 hours, with a mean AUC of 0.98 (SD 0.003). The distribution of the PR curve approached the ideal upper bound, indicating effective identification of subtle injury regions during late-stage tissue repair.

At the 24-hour time point, the model maintained strong classification capability with a mean AUC of 0.97 (SD 0.005), indicating high generalizability for evaluating early therapeutic responses. Even during the acute injury phase at 6 hours, the model demonstrated robust discrimination, achieving a mean AUC of 0.91 (SD 0.012) and consistently high precision in the PR curve. This implies that the transformer is effective at identifying typical necrotic and apoptotic features even in early-stage pathology.

Overall, the model exhibited stable classification performance across all 3 time points, supporting its applicability to both static image discrimination and the analysis of complex morphological variations across different stages of time series tasks.

### Model Interpretability and Biological Consistency of Attention Maps

**Figure 9.**
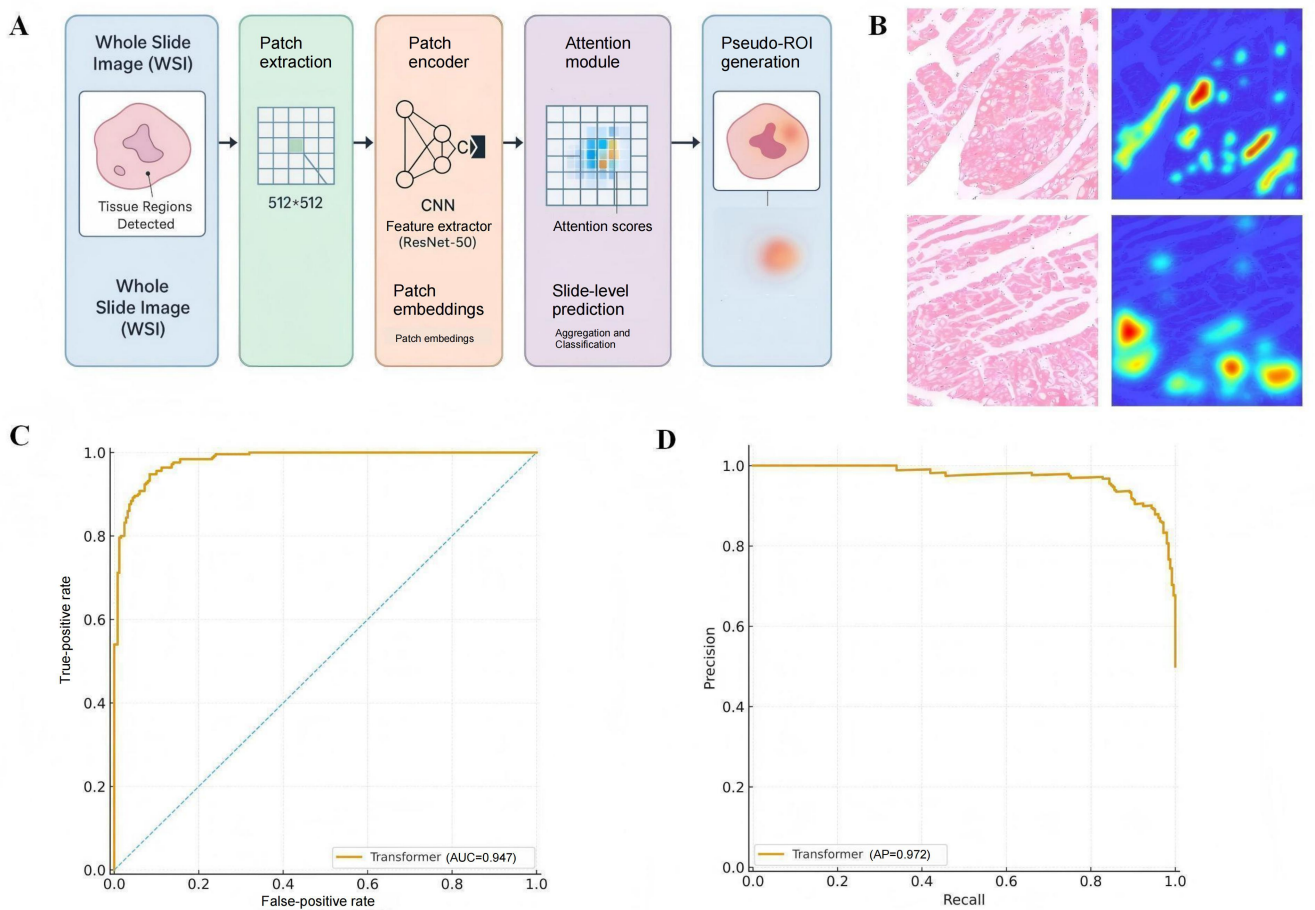
Workflow and performance of the MIL-based weakly supervised framework. (A) Schematic diagram of the weakly supervised learning pipeline. Whole-slide images (WSIs) are partitioned into 512×512-pixel tiles and assigned slide-level labels based on treatment group and reperfusion time. Using the CLAM framework, the model applies an attention mechanism to automatically generate pseudo-regions of interest (ROIs) and learn pathological discriminative features. (B) Representative H&E image overlaid with the attention heatmap. Red regions denote high-importance areas identified by the model. (C) Receiver operating characteristic (ROC) curve of the weakly supervised transformer classifier, showing an area under the curve (AUC) of 0.947. (D) Precision-recall (PR) curve illustrating the balance between precision and recall across varying thresholds. The attention-derived pseudo-ROIs generated from this workflow are subsequently used to guide model pretraining.

To further evaluate the interpretability and biological plausibility of the transformer model in histopathological image classification, we conducted a weakly supervised analysis based on an MIL framework (Figure 9). Specifically, we adopted the CLAM architecture, in which each myocardial H&E WSI was partitioned into 512×512 pixel patches and trained under weak supervision using reperfusion time points and treatment group labels. Through its attention mechanism, the model automatically generated pseudo-ROIs, highlighting diagnostically relevant areas without requiring pixel-level annotations.

The resulting attention heatmaps showed high spatial concordance with key histopathological regions, including necrotic border zones and inflammatory infiltration areas. Highly weighted red regions consistently aligned with structurally compromised zones or areas with dense inflammatory cell accumulation, indicating that the features learned by the model carried clear biological meaning (Figure 9B). The weakly supervised transformer classifier demonstrated strong performance on the held-out test set, achieving a mean AUC of 0.947 (SD 0.006) on the ROC curve (Figure 9C), and its precision-recall curve showed balanced high precision and recall (Figure 9D).

Collectively, these results demonstrate that the transformer model not only outperforms other deep learning architectures in quantitative metrics but also provides intrinsically interpretable outputs. The attention mechanism consistently identified pathologically relevant structures, enhancing transparency, supporting biological consistency, and strengthening the translational potential of transformer-based models in medical histopathology.

### Model Validation and Consistency Analysis

**Figure 10.**
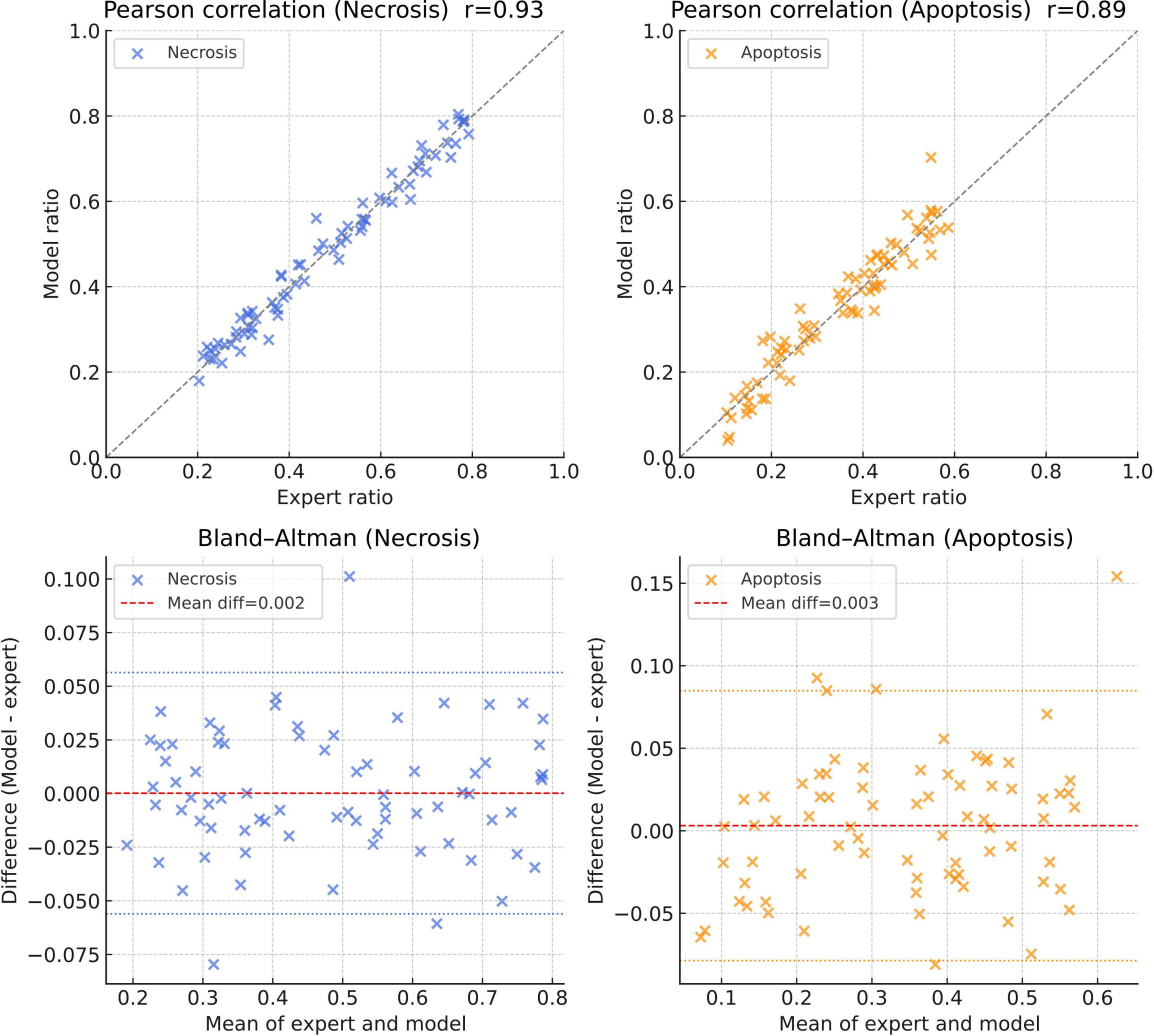
Consistency analysis between model outputs and pathologist measurements for necrotic and apoptotic regions. Top row: Pearson correlation analyses comparing model-predicted necrotic and apoptotic areas with manual measurements by expert pathologists. Strong linear positive correlations were observed for both necrosis (*r*=0.93) and apoptosis (*r*=0.89), with all *P*<.001. The dashed line represents the line of perfect agreement. Bottom row: Bland-Altman plots assessing agreement between model predictions and expert annotations. The mean differences were 0.002 for necrotic regions and 0.003 for apoptotic regions, with 95% limits of agreement within +5% and −5% and no systematic bias detected. Blue and orange points represent necrotic and apoptotic samples, respectively. These results demonstrate a high degree of concordance between the transformer model and expert assessments, confirming the model’s reliability and clinical consistency in identifying myocardial injury regions.

To evaluate the reliability of the transformer model in detecting dynamic tissue changes associated with MIRI, we conducted expert pathological review and statistical consistency analyses using an independent dataset (Figure 10). Two senior cardiovascular pathologists independently measured the proportions of necrotic and apoptotic areas under blinded conditions (25 images per time point). These manual measurements were then compared with model-derived outputs. The results demonstrated strong concordance between human assessments and model predictions. The mean Pearson correlation coefficients (*r*) for necrotic and apoptotic area ratios were 0.93 (SD 0.02) and 0.89 (SD 0.03), respectively (both *P*<.001), indicating a high degree of alignment. Bland-Altman analyses further supported this agreement, with mean differences of 0.002 for necrotic regions and 0.003 for apoptotic regions, 95% limits of agreement confined within +5% and −5%, and no evidence of systematic bias.

The model’s temporal recognition capability was further validated using the independent BioImage Archive dataset (S-EPMC9426742). The temporal trajectories of necrotic and apoptotic area reduction across reperfusion time points closely matched the patterns observed in the local validation cohort. These results confirm that the transformer model reliably captures biologically meaningful, time-dependent tissue changes following reperfusion. The dynamic differences detected by the model reflect authentic pathological progression rather than algorithmic artifacts, underscoring its stability, interpretability, and suitability for longitudinal histopathological assessment.

### Concordance Analysis Between Model Outputs and Expert Annotations

**Figure 11.**
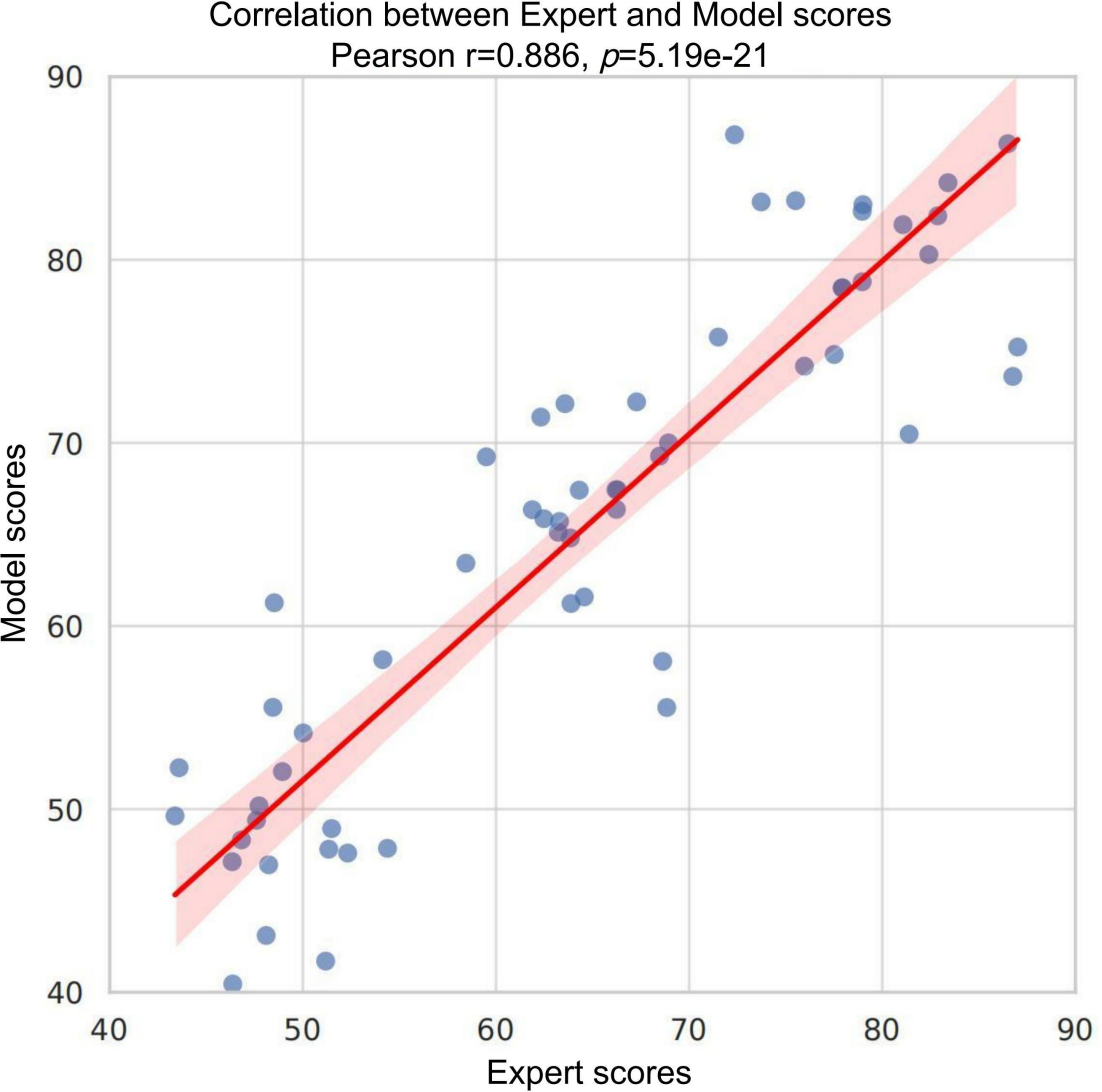
Linear correlation between model predictions and expert pathological scores. The scatter plot illustrates the correspondence between transformer model predictions and expert manual assessments. The red line represents the linear regression trend, with the shaded area indicating the 95% CI. The Pearson correlation coefficient is 0.886 with a *P* value of 5.19×10^-21^, indicating a strong positive correlation and validating the model’s consistency and reliability in assessing tissue injury severity.

To further validate the reliability and interpretability of the transformer model in recognizing myocardial tissue injury, we performed a pairwise comparison between model-generated scores and pathologist-assigned expert scores, and visualized the results using a correlation scatter plot (Figure 11). Statistical analysis revealed a significant positive linear correlation between the 2 sets of scores (mean Pearson correlation coefficient *r*=0.886, SD 0.018; *P*=5.19×10^−21^). The regression line exhibited a stable slope with a narrow 95% CI, indicating a high level of agreement between the model outputs and expert assessments.

The results confirm the reliability of the model predictions from a statistical perspective and demonstrate strong trend-level consistency with expert assessments from a clinical standpoint. The findings further support the potential of the transformer model as an auxiliary evaluation tool in practical applications by providing a structured and quantifiable basis for interpreting the severity of myocardial injury.

## Discussion

MIRI represents a critical pathological process in the treatment of cardiovascular diseases and is typically characterized by marked cardiomyocyte apoptosis and necrosis, which directly influence the efficacy of clinical interventions and patient prognosis [30,31]. Traditional pathological assessment depends heavily on manual slide interpretation and is limited by subjectivity, low efficiency, and poor reproducibility. In recent years, rapid advances in AI, particularly deep learning–based medical image recognition, have highlighted the potential of automated H&E-stained tissue analysis to improve the quality and consistency of histopathological evaluation. In this context, this study constructed a large-scale H&E image dataset encompassing multiple intervention groups and reperfusion time points. Performance comparisons across 8 mainstream deep learning models for myocardial injury recognition identified the transformer-based architecture as the optimal option. An integrated framework for AI-assisted histopathological assessment was also established, combining image recognition, trend quantification, and visual output. The framework provides a novel technical pathway to support clinical interpretation of myocardial pathology and offers a foundation for more standardized and quantitative evaluation in translational cardiovascular research.

Deep learning has seen increasing application in cardiovascular pathology image analysis in recent years. However, most studies remain focused on static image recognition simultaneously, with typical tasks including apoptotic cell detection, fibrotic region segmentation, or immunohistochemical image classification. These investigations predominantly rely on CNN-based models or their variants, focusing on local feature extraction capabilities [32]. Some studies have explored the use of GANs for image augmentation or used LSTMs for processing temporal data, but spatiotemporal integration at the tissue level remains largely underdeveloped [33,34]. In contrast, this study incorporates diverse therapeutic contexts and systematically introduces the temporal dimension. By leveraging the strengths of the transformer architecture in spatial representation and long-range dependency modeling, high-precision recognition of histopathological progression across multiple reperfusion time points was achieved. Model outputs are further validated against expert annotations to ensure interpretability and clinical consistency. The proposed approach addresses limitations of prior work that emphasized classification accuracy without adequate clinical benchmarking and advances the application of AI from static image recognition toward dynamic pathological interpretation.

In this study, both human-derived and classical rodent MIRI sections were incorporated during the model training phase, primarily based on the fact that this pathological process exhibits highly consistent histological endpoint features across different species. Nevertheless, interpretations related to clinical relevance in this work are primarily grounded in the model’s performance on human samples. Animal-derived samples are mainly leveraged to enhance the model’s ability to learn generalizable pathological phenotypes of myocardial reperfusion injury and to improve its robustness and stability of generalization.

It is worth noting that the strong performance of the transformer model in this study arises not only from its global receptive field and robust spatial modeling capacity but also from its compatibility with the complex spatial textures and gradual color transitions that frequently characterize myocardial injury images. Although some studies report that GANs perform well in image augmentation and feature completion, the results show that GANs yield only modest performance gains in this task, suggesting greater utility in contexts involving severe class imbalance or limited sample availability. Heatmap analysis further demonstrates a high degree of correspondence between the attention regions generated by the transformer model and the pathological zones of interest. In the antioxidant treatment group in particular, low activation intensity in blue aligns with reduced apoptosis and necrosis, reflecting strong agreement with histopathological findings. Such spatial concordance strengthens model interpretability and provides empirical support for the concept of biological consistency, in which AI outputs correspond to biologically meaningful patterns. These findings collectively highlight the potential of deep learning to assist with mechanistic inference in MIRI.

From a clinical perspective, the proposed AI framework demonstrates broad adaptability and strong potential for translation into routine practice. Integration into digital pathology platforms enables automated identification and quantitative assessment of MIRI on histological sections, thereby supporting pathologists in interpretation, particularly in high-throughput or repetitive evaluation environments. The framework also provides a quantitative basis for drug screening, therapeutic efficacy assessment, and prognostic evaluation, offering standardized and objective data that can enhance cardiovascular research. Visual outputs generated by the model, including heatmaps and temporal trend plots, can be applied in research, training, and educational settings to improve understanding of pathological progression among junior clinicians and investigators. Given the model’s high classification accuracy across treatment groups and its ability to characterize temporal dynamics, broader adoption in preclinical studies and clinical research is recommended to support mechanistic investigation and to improve the efficiency and quality of precision medicine in cardiovascular disease.

Although the study has produced meaningful findings, several limitations remain. First, model training and validation relied on publicly available datasets. These datasets offer relatively large sample sizes and stable quality, yet they do not fully capture common clinical challenges, such as sectioning artifacts, staining variability, or scanning-induced noise, which may affect generalizability in routine clinical environments. Second, this analysis is restricted to static histopathological images and has not incorporated multimodal information from serial tissue sections, biochemical indicators, electrocardiogram data, or other physiological time series measurements. As a result, the relationship between microscopic pathology and macroscopic physiological processes has not been comprehensively explored. Third, although the transformer architecture achieves high classification accuracy, its substantial parameter count and slower inference speed require considerable computational resources, which may hinder real-time deployment on edge devices or remote platforms. These limitations indicate that future research should aim to enhance dataset diversity, develop lightweight models suitable for broader deployment, and promote deeper integration of multimodal clinical information.

Future research can progress in several directions. First, expanding the dataset to incorporate clinical samples from multiple hospitals, imaging platforms, and slide preparation protocols will strengthen cross-center robustness and improve generalizability. Second, integrating histological image data with multiomics biomarkers, including genomic, transcriptomic, and serological information, may support the development of end-to-end AI systems with deeper mechanistic relevance. Third, incorporating explainable AI techniques to investigate the causal links between model attention regions and pathological alterations will promote greater verifiability, interpretability, and clinical trust. Fourth, in terms of model optimization, approaches such as model compression and knowledge distillation may enhance computational efficiency and facilitate deployment in edge computing environments or real-time clinical workflows. In addition, the current framework can be extended to the analysis of other tissue injury contexts, including cerebral ischemia, liver fibrosis, and pulmonary damage, thereby contributing to the construction of a broader AI framework for histopathological evaluation across diverse disease domains.

In conclusion, the study constructs a large-scale dataset of myocardial H&E-stained images and provides a systematic comparison of 8 deep learning models for recognizing reperfusion injury. The results show that transformer-based models consistently achieve superior performance, with clear advantages in interpreting dynamic pathological changes and evaluating treatment responses. The findings broaden the application of AI in myocardial injury analysis and establish a scalable AI-assisted evaluation framework. With high classification accuracy, strong temporal trend tracking, and close agreement with expert assessments, the proposed approach demonstrates both scientific value and clinical applicability. Although certain technical and methodological limitations remain, the study provides a solid foundation for the development of standardized, high-throughput, and mechanism-oriented AI systems in digital pathology and offers robust technological support for future advances in precision medicine (Multimedia Appendix 6).

## Supplementary material

10.2196/80403 Multimedia Appendix 1 Comparative illustration of traditional slide reading and AI-assisted evaluation methods.

10.2196/80403 Multimedia Appendix 2 Composition and class distribution of the histopathological image dataset.

10.2196/80403 Multimedia Appendix 3 Supplementary protocol P1 and code S1.

10.2196/80403 Multimedia Appendix 4 Schematic diagrams of 8 AI algorithm models. The figure presents representative architectures of the following deep learning models: (A) convolutional neural networks, (B) recurrent neural networks, (C) long short-term memory networks, (D) autoencoders, (E) generative adversarial networks, (F) graph convolutional networks, (G) transformer-based attention models, and (H) variational autoencoders.

10.2196/80403 Multimedia Appendix 5 Comparison of deep learning models in terms of training stability, convergence speed, and inference efficiency.

10.2196/80403 Multimedia Appendix 6 Integrated workflow of myocardial ischemia-reperfusion injury (MIRI) tissue recognition and therapeutic evaluation based on transformer model. This figure illustrates the complete research workflow from data acquisition to model training, image recognition, therapeutic evaluation, and expert validation. Initially, hematoxylin and eosin–stained images of MIRI are collected and standardized. Eight deep learning models are compared to identify the optimal architecture, with the transformer model selected for its superior performance. The model outputs include quantifying damaged regions, time-dependent progression trends, and confidence heatmaps, which are further validated against expert scores, demonstrating the model's broad applicability in cardiovascular pathology research and clinical decision support.

## Abbreviations

AI: artificial intelligence
AUC: area under the curve
CLAM: clustering-constrained attention multiple-instance learning
CNN: convolutional neural network
DCGAN: Deep Convolutional Generative Adversarial Network
GAN: generative adversarial network
GCN: graph convolutional network
H&E: hematoxylin and eosin
IS: inception score
LSTM: long short-term memory network
MI: myocardial infarction
MIL: multiple-instance learning
MIRI: myocardial ischemia-reperfusion injury
PR: precision-recall
RNN: recurrent neural network
ROC: receiver operating characteristic
ROI: region of interest
SSIM: Structural Similarity Index
VAE: variational autoencoder
WSI: whole-slide image
