# Supplementary figures and images for: Histopathological Assessment of Myocardial Ischemia-Reperfusion Injury Using Transformer-Based Artificial Intelligence: Model Comparison Study
Source: JMIR Med Inform. 2026 Jun 4;14:e80403. doi: 10.2196/80403 (PMC13235984; doi:10.2196/80403)

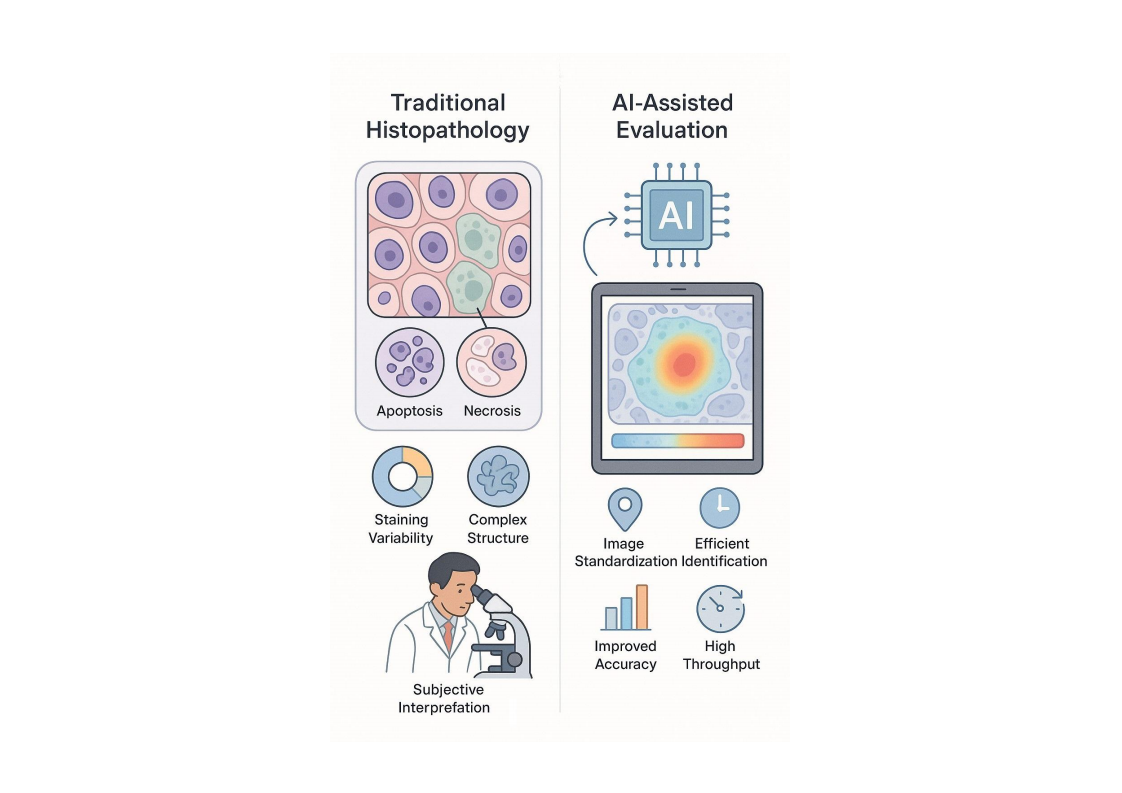

Supplement: Multimedia Appendix 1 [file medinform-v14-e80403-s001.png]

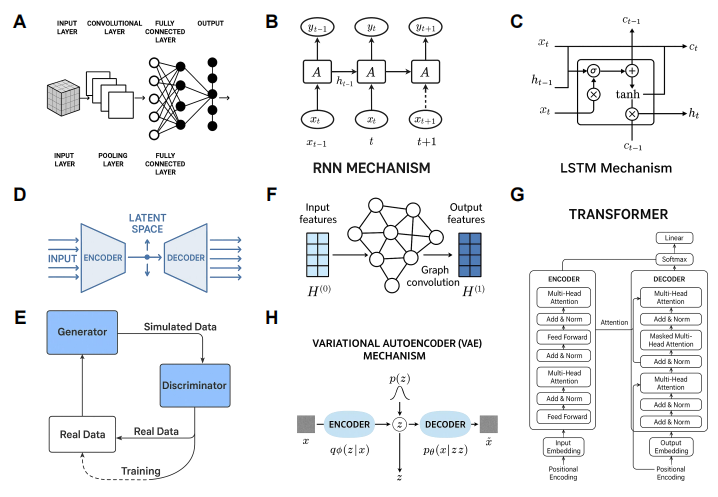

Supplement: Multimedia Appendix 4 [file medinform-v14-e80403-s004.png]
