# Supplementary material for: Histopathological Assessment of Myocardial Ischemia-Reperfusion Injury Using Transformer-Based Artificial Intelligence: Model Comparison Study
Source: JMIR Med Inform. 2026 Jun 4;14:e80403. doi: 10.2196/80403 (PMC13235984; doi:10.2196/80403)
Supplement: Multimedia Appendix 5 [file medinform-v14-e80403-s005.docx]

**Appendix 2. Comparison of Deep Learning Models in Terms of Training Stability, Convergence Speed, and Inference Efficiency.**

| **Model** | **Training Stability (Loss SD)** | **Convergence Epochs** | **Inference Time (ms)** |
| --- | --- | --- | --- |
| CNNs | 0.125 | 15 | 10 |
| RNNs | 0.194 | 19 | 59.6 |
| LSTMs | 0.168 | 14 | 40.9 |
| Autoencoders | 0.152 | 33 | 40.6 |
| GANs | 0.099 | 32 | 10.4 |
| GCNs | 0.099 | 13 | 11.2 |
| VAEs | 0.087 | 23 | 36.2 |
| Transformers | 0.184 | 17 | 30 |
