# Supplementary material for: Histopathological Assessment of Myocardial Ischemia-Reperfusion Injury Using Transformer-Based Artificial Intelligence: Model Comparison Study
Source: JMIR Med Inform. 2026 Jun 4;14:e80403. doi: 10.2196/80403 (PMC13235984; doi:10.2196/80403)
Supplement: Multimedia Appendix 6 [file medinform-v14-e80403-s006.pdf]

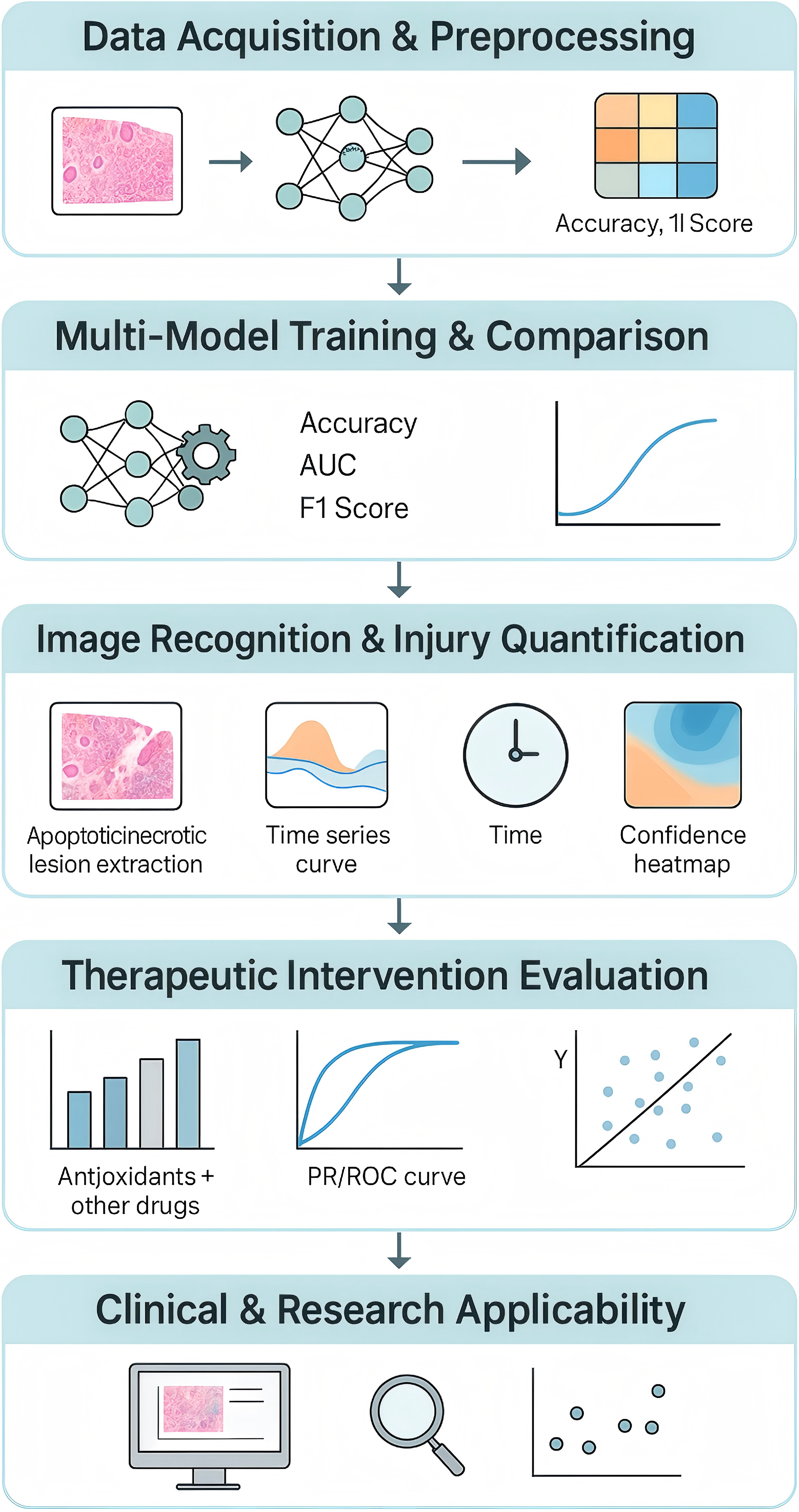
Data Acquisition & Preprocessing
Accuracy, 1l Score
Multi-Model Training & Comparison
Accuracy
AUC
F1 Score
Image Recognition & Injury Quantification
Apoptoticinecrotic lesion extraction
Time series curve
Time
Confidence heatmap
Therapeutic Intervention Evaluation
Antjoxidants + other drugs
PR/ROC curve
Y
Clinical & Research Applicability
